# Cytoplasm-nucleus shuttling of TET2: an intrinsic brake in colorectal cancer progression

**DOI:** 10.1038/s41419-026-08418-5

**Published:** 2026-01-28

**Authors:** Changpeng Li, Fei Meng, Jingcai He, Linna Dong, Yuexian He, Qing Guo, Kerou Zeng, Yanhua Wu, Haofei Ge, Shiyu Chen, Tingting Yang, Yusheng Zhou, Yulu Wang, Lin Liu, Qiwen Ren, Meiai He, Hao Sun, Lining Liang, Lin Guo, Xiaolin Li, Jiahong Hong, Zhenhua Huang, Hui Zheng

**Affiliations:** 1 Guangdong Provincial Key Laboratory of Stem Cell and Regenerative Medicine, Guangdong-Hong Kong Joint Laboratory for Stem Cell and Regenerative Medicine, GIBH-CUHK Joint Research Laboratory on Stem Cell and Regenerative Medicine, Guangzhou Institutes of Biomedicine and Health, Chinese Academy of Sciences, Guangzhou, China; 2 Centre for Regenerative Medicine and Health, Hong Kong Institute of Science & Innovation, Chinese Academy of Sciences, Hong Kong SAR, China; 3 Key Laboratory of Biological Targeting Diagnosis, Therapy and Rehabilitation of Guangdong Higher Education Institutes, The Fifth Affiliated Hospital of Guangzhou Medical University, Guangzhou, China; 4 University of Chinese Academy of Sciences, Beijing, China; 5 Joint School of Life Sciences, Guangzhou Medical University, Guangzhou, China; 6 Department of Oncology, Nanfang Hospital, Southern Medical University, Guangzhou, China

**Keywords:** Cell migration, Cancer

## Abstract

Colorectal Cancer (CRC) progression is a complex and dynamic process closely linked to TET2-mediated DNA demethylation. Distinct from our previous study on TET2 nuclear loss, which can be observed in the whole tumor progression process, the nuclear increase of TET2 was only observed in tumors at the beginning of metastasis. In addition, cells with nuclear TET2 were located at the bottom of the mucosa, which is the invasion front of CRC. All of these results suggested crucial roles of TET2 nuclear increase during tumor progression. Mechanistically, epithelial-mesenchymal transition (EMT) and the activation of the WNT pathway, which is normally recognized as tumor promotion events, were shown to correlate with the cytoplasm-nucleus shuttling of TET2, which is associated with tumor suppression. Nuclear TET2, in turn, mitigated further EMT and WNT activation, suggesting a negative feedback loop between TET2 and the EMT/WNT pathway. Such a negative feedback loop was further supported by single-cell RNA sequencing (scRNA-seq) analysis of both the CRC progression models and the clinical CRC samples. Together, these findings indicate that the tumor inhibition role of EMT/WNT pathway and TET2 is an intrinsic brake on cancer progression, which represents a potential therapeutic target for CRC.

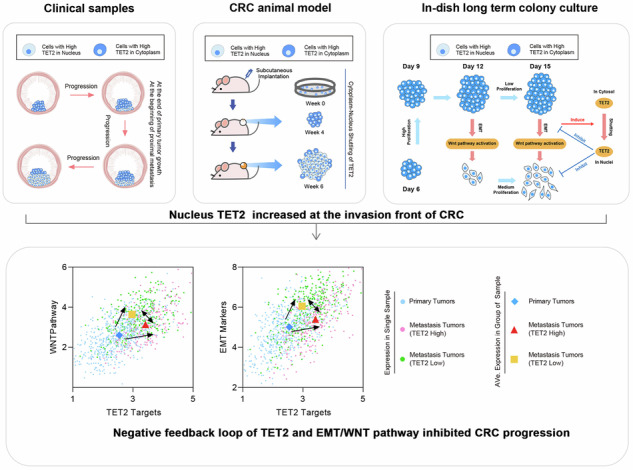

## Introduction

Ten-eleven translocation methylcytosine dioxygenases 2 (TET2) catalyze the 5mC to 5-carboxylcytosine (5caC) [1, 2], and the deregulation of TET2 is associated with severe consequences, such as functional failure, as well as the initiation of diseases, including cancer [3].

EMT is normally considered as an important cell fate transition process of CRC, which promotes tumor progression [4]. Notably, certain metastatic subpopulations in pancreatic and breast carcinomas [5, 6] demonstrate EMT irrelevant dissemination, collectively revealing the complexity of EMT in tumor progression. In CRC, the WNT pathway is also activated through nuclear accumulation of β-catenin, which is also a critical regulator and marker of EMT [7]. EMT and the activation of the WNT pathway often coincide during the progression of solid tumors [8, 9]. These pathways play crucial roles in the late-stage of tumor progression by regulating processes such as the development and maintenance of invasive potential, stemness, disruption of cell-cell adhesion, and resistance to therapy [10].

In recent years, many compounds have been developed to target the EMT/WNT pathway for cancer treatment. However, few have succeeded, highlighting the complexity of these pathways [11]. In this study, we observed a tumor-inhibitory effect of EMT/WNT pathway by promoting the cytoplasm-nucleus shuttling of TET2, which inhibited the progression of CRC. And the EMT/WNT pathway formed a negative feedback loop with TET2, making TET2 an intrinsic brake against CRC progression.

## Results

### Impact of TET2 localization on survival and progression of CRC patients

**Fig. 1:**
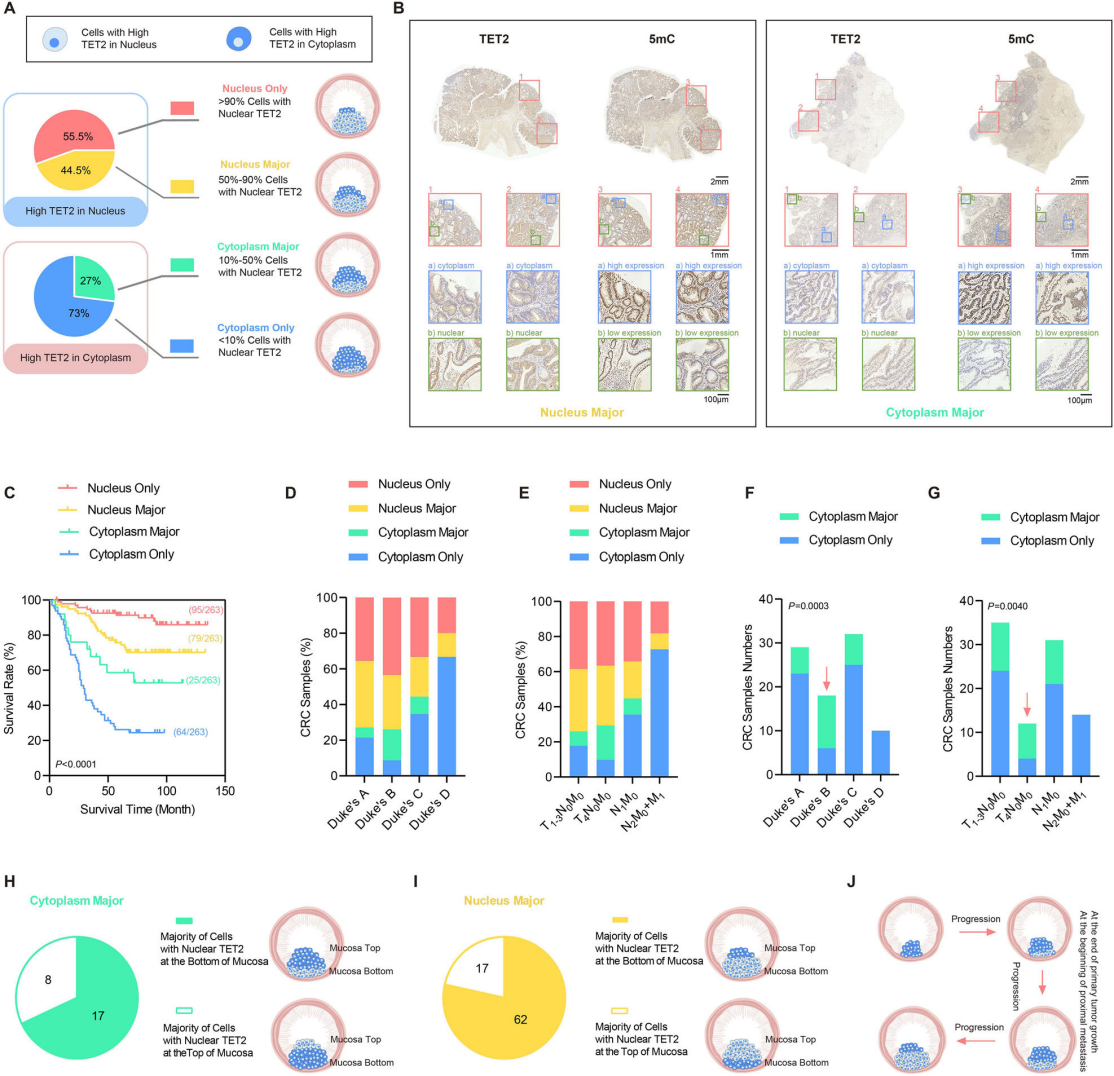
Mosaic localization pattern of TET2 is observed in CRC tissues. **A** The schematic diagram shows: samples of TET2 with “nucleus only” and “nucleus major” were categorized from the previously classified “High TET2 in the nucleus” samples; similarly, samples of TET2 with “cytoplasm only” and “cytoplasm major” were segmented from the earlier identified “High TET2 in the cytoplasm” samples. **B** Representative IHC images of TET2 and 5mC in serial sections for CRC samples categorized into different types. Note that “cytoplasm only” and “nucleus only” were already shown in our previous study. **C** Kaplan–Meier survival plots for the four indicated groups of all 263 patients. All 263 samples were classified with Duck’s (**D**) and TNM (**E**) staging to analyze the relationship between staging and the four indicated groups of samples. The percentage of “cytoplasm major” patients increased in the indicated Duke’s stage B (**F**) and T_4_N_0_M_0_ stage (**G**). The distribution of cells with nuclear TET2 at the bottom and the top of the mucosa was summarized in the “cytoplasm major” (**H**) and the “nuclear major” (**I**) groups. **J** A schematic illustration of the cytoplasm-nucleus shuttling of TET2 localization during CRC progression. Additional statistical information was provided in Table S3.

In the current study, samples were divided into four categories based on the percentage of cells showing nuclear TET2 and low levels of 5mC. Samples with over 90% nuclear TET2, 50–90% nuclear TET2, 10–50%nuclear TET2, and below 10% nuclear TET2 were respectively classified as the “nucleus only”, “nucleus major”, “cytoplasm major”, and the “cytoplasm only” group. Correspondingly, 5mC levels varied across these groups, reflecting the shift in TET2 subcellular distribution and activity (Fig. 1A, B and Table S1).

Consistent with our previous results [12], higher levels of nuclear TET2 were correlated with better survival of the corresponding CRC patients. The “nucleus only” group exhibited better survival than the “nucleus major” group, and the “cytoplasm major” group exhibited better survival than the “cytoplasm only” in both cohorts of patients enrolled in this study (Figs. 1C and S1A, B). To further determine the change of the localization of TET2 in tumor progression, we checked the subcellular distribution pattern of TET2 across different Duke’s stages. We observed that as Duke’s stage advanced from A to D, the proportions of samples categorized as the “nucleus only” and “nucleus major” decreased. Conversely, the proportions of samples classified as the “cytoplasm only” and “cytoplasm major” increased (Figs. 1D and S1C, D). A similar phenomenon was also observed with TNM staging (Figs. 1E and S1E, F). All these data indicated that the nuclear localization of TET2 decreased during the tumor progression process.

Next, we analyzed the nuclear loss of TET2 by comparing the ratio of samples between the “nucleus only” and “nucleus major” groups across different tumor stages, as nuclear loss is more likely to occur in samples with predominantly nuclear TET2. Unfortunately, the nuclear loss of TET2 did not show a significant preference across CRC stages (Fig. S1G, H). Conversely, we investigated the cytoplasm-nucleus shuttling of TET2 in samples classified as the “cytoplasm major” and “cytoplasm only”. As shown in Fig. 1F, G, significantly more samples in the “cytoplasm major” groups were observed at Duke’s stage B and T_4_N_0_M_0_ stage. These results indicated a slight increase in TET2 nuclear localization in this period of tumor growing, but the overall nucleus TET2 was decreased, as we can see the highest stages of CRC exhibit lowest level of nucleus TET2 (Fig. 1F, G).

CRC cancer cells at Duke’s stage B and T_4_N_0_M_0_ are typically at a stage where the primary tumor has reached its local growth limit, and metastasis begins [13]. We analyzed the localization of cells with nuclear TET2 in these samples. Cells with nuclear TET2 were more frequently found at the bottom of the mucosa rather than the top in samples from the “cytoplasm major” group (Fig. 1H and S1I). In addition, similar phenomena were observed with samples of the “nucleus major” group (Figs. 1I and S1I). Since CRC tumor cell invasion typically occurs in the bottom area of the mucosa, which is associated with conditions such as nutrient and oxygen deprivation that initiate EMT [14], it is reasonable to suggest that the cytoplasm-nucleus shuttling of TET2 occurs at the end of primary tumor growth or at the onset of proximal metastasis (Fig. 1J). Finally, multivariate Cox regression analysis demonstrated that TET2 acts as an independent prognostic factor of CRC survival (Fig. S2).

### Dynamics of TET2 subcellular localization during tumor growth

**Fig. 2:**
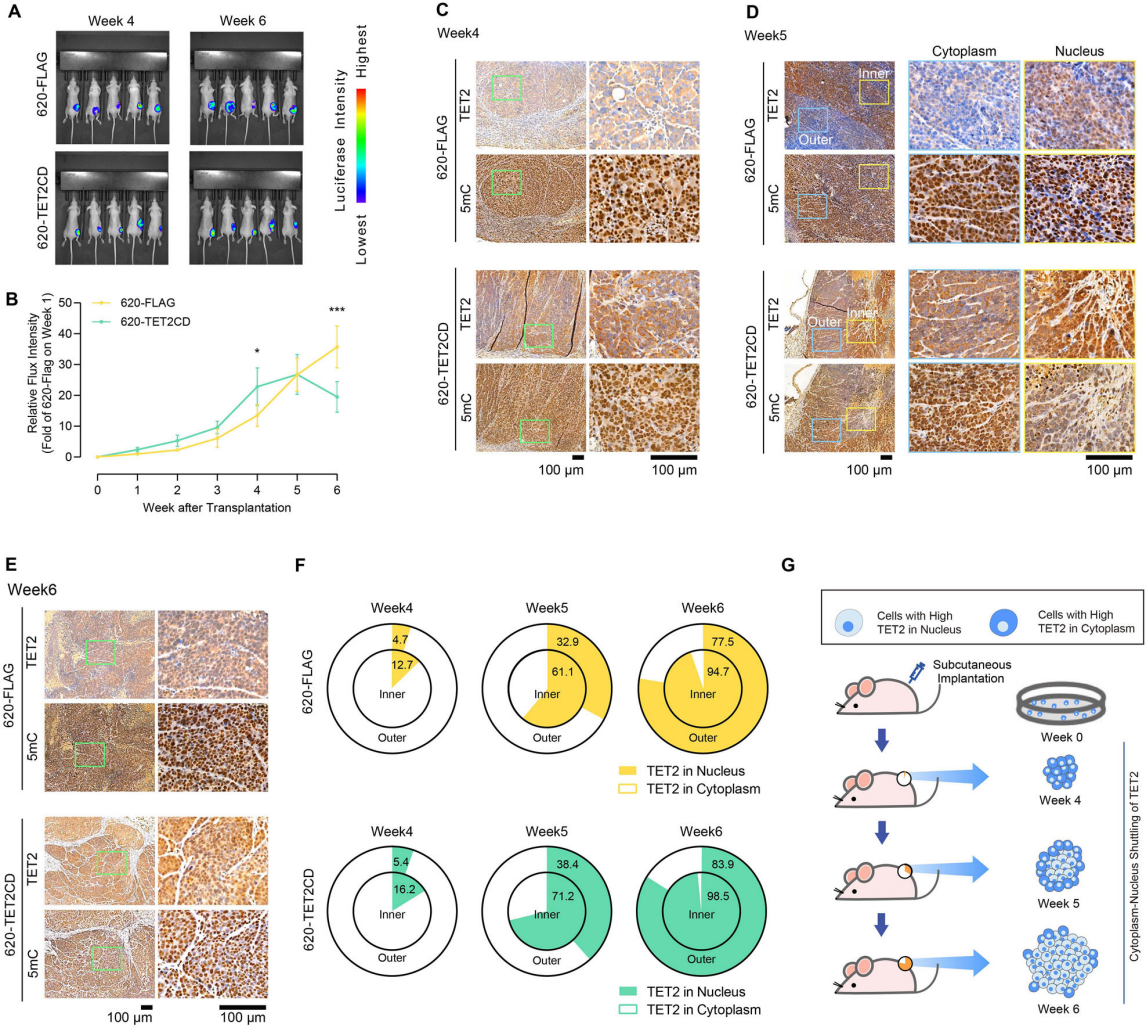
The cytoplasm-nucleus shuttling of TET2 was observed during CRC progression in nude mice. **A**, **B** SW620 cells over-expressing FLAG or TET2CD were transplanted into nude mice with living imaging (**A**). Tumor sizes were monitored and compared at the indicated time points (**B**). Note that only living cells were measured. IHC was used to determine the expression and localization of TET2 and the level of 5mC at week 4 (the early stage) (**C**), week 5 (the middle stage) (**D**) and week 6 (the late stage) (**E**). **F** The cytoplasm-nucleus shuttling of TET2 in the inner and outer of tumor mass were summarized at week4, week5, and week6. **G** The cytoplasmic-nuclear shuttling of TET2 during tumor growth of SW620 cells in nude mice is summarized in a schematic diagram. Additional statistical information was provided in Table S3.

To confirm the cytoplasm-nucleus shuttling of TET2 during the progression of CRC, we subcutaneously transplanted wild-type SW620 cells overexpressing an empty FLAG-vector (SW620-FLAG) and cells overexpressing the catalytic domain of TET2 (SW620-TET2CD), both of which exhibit cytoplasmic TET2 [12], into nude mice (Fig. 2A).

During the initial four weeks of transplantation, the overexpression of TET2CD did not suppress the growth of SW620 cells in nude mice (Fig. 2A, B). The subcellular localization analysis at week 4 disclosed that both endogenous and exogenous TET2 were predominantly located in the cytoplasm, which was in line with the strong IHC signals against 5mC (Fig. 2C). At week 5, an increase in nuclear TET2 and a decrease in 5mC signal were observed in mice transplanted with both SW620-FLAG and SW620-TET2CD cells (Fig. 2D). At week 6, nuclear TET2 area expanded and surpassed cytoplasmic TET2. With the increase in nuclear TET2, the overexpressed TET2CD acquired the ability to induce DNA demethylation and inhibit tumor growth, as evidenced by the smaller tumor sizes in SW620-TET2CD-transplanted mice (Fig. 2B) and the decreased IHC signals against 5mC (Fig. 2E).

Importantly, the nuclear translocation of TET2 appears to initially occur in the inner regions of tumor tissues (Fig. 2C–F). These inner tumor areas in nude mice models have long been known to experience hypoxia and nutrient deprivation during the rapid growth of the tumor mass [15]. The cytoplasm-nucleus shuttling of TET2 may thus be closely associated with hypoxia and nutrient scarcity. In conclusion, the cytoplasm-nucleus shuttling of TET2 was observed during the growth of SW620 tumors in nude mice (Fig. 2G).

### EMT/WNT pathway activation is associated with cytoplasm-nucleus shuttling of TET2 during long-term culture

Since SW620 cells exhibit high expression levels of E-Cadherin and strong cell-cell interactions [12], they initially grow as individual colonies in a spherical shape with multiple layers of cells during the early culture period (day 0–9) in a petri dish in vitro. As the culture time increases, the cells inside colonies migrate outward and acquire a mesenchymal morphology by day 12–15 distinct from SW480 (Fig. S3A). These colony characteristics of SW620 cells closely resemble the real tumor growth dynamics. To mimic tumor growth in vitro, we cultured SW620 colonies for an extended period (long-term culture assay, LTC) of 15 days (Fig. S3A).

**Fig. 3:**
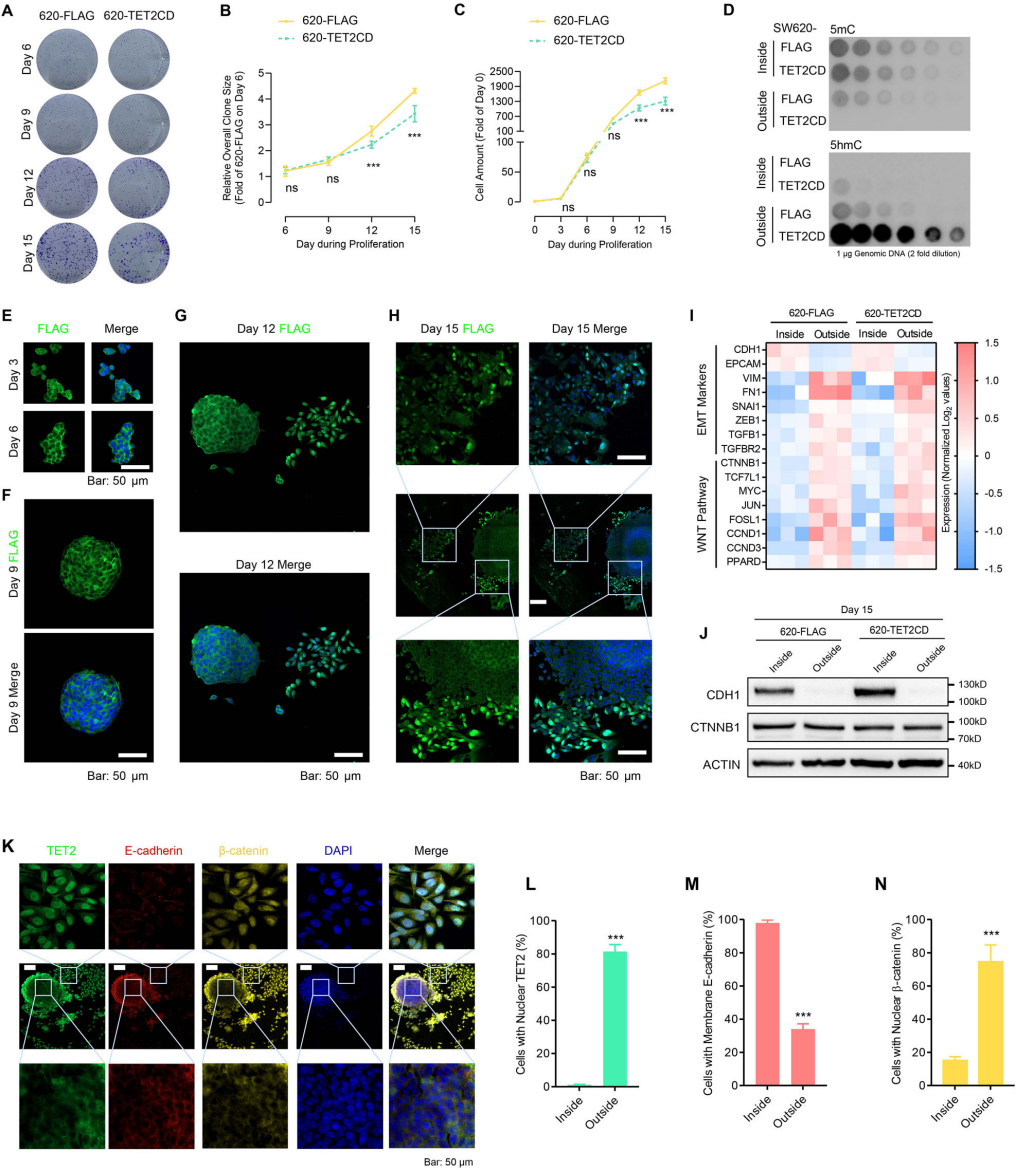
The cytoplasm-nucleus shuttling of TET2 was observed in SW620 LTC. **A**–**C** SW620 single cell colonies were cultured for 15 days without passage stained with crystal violet (**A**). The overall colony size (**B**) and the amounts of cells (**C**) were summarized. **D** The activity of TET2 in cells inside and outside the colonies was measured with 5mC/5hmC Dotblot on Day 15. IF was conducted at day 3 (**E**), 6 (**E**), 9 (**F**), 12 (**G**) and 15 (**H**) to indicate the localization of TET2CD. **I** The expression of selected markers in RNA-seq analysis. **J** Immunoblotting of CDH1 and CTNNB1 in cells inside and outside the colonies. **K**–**N** IF was performed to determine the localizations of TET2, CDH1 and CTNNB1 in cells inside and outside the colonies on day 15 (**K**). The cells with nuclear TET2 (**L**), membrane E-cadherin (**M**), and nuclear β-catenin (**N**) were summarized. Additional statistical information was provided in Table S3.

During the initial phase of SW620 colony culture (day 0-9), TET2CD did not significantly inhibit the growth of SW620 (Fig. 3A–C). However, progressively enhanced suppressive effects of TET2CD on SW620 growth were observed from day 9 to day 15 (Fig. 3A–C). Subsequent Dot Blot analysis demonstrated significant DNA demethylation induced by TET2 in the cells outside the colonies, indicating the activation of TET2 in these cells (Fig. 3D). These findings collectively suggest the occurrence of cytoplasm-nucleus shuttling of TET2 during the later stage of SW620 LTC.

To confirm the cytoplasm-nucleus shuttling of TET2, we monitored its subcellular localization during SW620-TET2CD LTC. Initially, TET2CD was predominantly localized in the cytoplasm during the days 0–9 of LTC (Fig. 3E, F). By days 12 and 15, the cells were categorized into two groups: those inside and outside the colonies. Inside cells are located within the cell colony with intense cell contact while outside cells are located at the edge or at the outside of cell colony with loosen cell contact. TET2CD remained in the cytoplasm in the cells inside the colonies, whereas it re-localized to the nucleus in the cells outside the colonies (Fig. 3G, H). Therefore, the cytoplasm-nucleus shuttling of TET2 occurred during the migration of cells out of the colonies. Note that in regular passage of SW620, TET2 remained in cytoplasm (Fig. S3B). Bulk RNA-seq analysis conducted on the cells inside and outside the colonies on day 15 confirmed the activation of the WNT pathway and EMT in the cells outside the colonies (Fig. 3I, GSE188329), which were closely correlated with the cytoplasm-nucleus shuttling of TET2. CDH1/CTNNB1 is widely accepted as the marker of mesenchymal status marker of EMT. So we also tested the protein level of CDH1 and CTNNB1 to illustrate the EMT status of cells. As shown in Fig. 3J–N, CDH1 expression decreased in outside cells. As for CTNNB1, its expression remained largely unchanged, while its nuclear localization increased.

SW480 cells, which are derived from the same patient as SW620, exhibit nuclear localization of TET2 [12, 16]. In SW480 cells, overexpression of TET2CD consistently suppressed cell growth at all time points during long-term colony culture (Fig. S3C–E) and induced significant DNA demethylation by day 15 (Fig. S3F). Furthermore, the expression of two known targets of TET2, *RORA*, and *SPARC* [12], was consistently increased in SW480-TET2CD cells during both the early and late stages of colony culture (Fig. S3G, H). However, SW620 cells overexpressing TET2CD showed increased expression of RORA and SPARC only in the late stage of LTC (Fig. S3I, J).

### Negative feedback between EMT and the nuclear shuttling of TET2

**Fig. 4:**
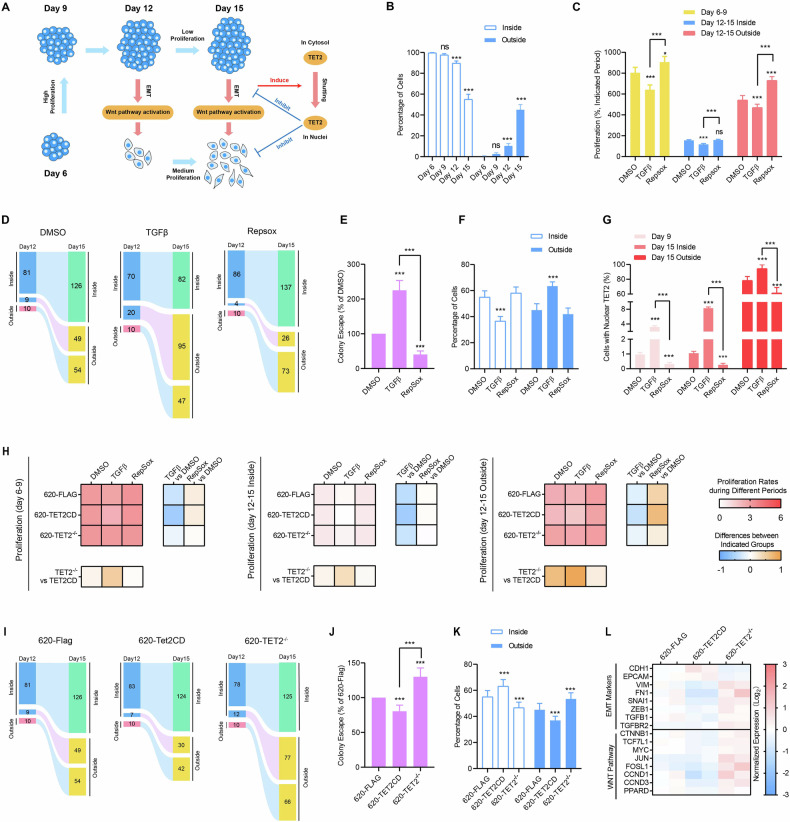
TET2 formed a negative feedback loop with the EMT/WNT pathway. **A** A schematic illustration of the LTC of SW620. **B** The percentages of cells inside and outsides the colonies at the indicated time points were counted and compared. (C-F) SW620 cells were treated with DMSO, 1 ng/mL TGF-β and 1 μM Repsox. The proliferation rates of cells were summarized in **C**. The migration of cells out of colonies was calculated based on the proliferation rates in (**C**) and presented in (**D**, **E**). The percentages of cells inside and outsides the colonies were summarized in (**F**). **G**, **H** SW620 cells with TET2CD overexpression and *TET2* knockout were treated with DMSO, 1 ng/mL TGF-β and 1 μM Repsox. The percentages of cells with nuclear TET2 were listed in (**G**), while the influences on cell proliferation were summarized in (**H**). TGF-β facilitated the cytoplasm-nucleus shuttling of TET2, and TET2 expression was essential for the ability of TGF-β to modulate cell proliferation, as shown by the heatmap. **I**–**L** Indicated cells were analyzed which shows that TET2 expression was essential for the migration of cells out of colonies **I**, **J** and was positively correlated with the percentages of cells inside the colonies (**K**). In addition, RNA-SEQ analysis shows that TET2CD overexpression suppressed the expression of EMT markers and targets in the WNT pathways, while *TET2* knockout functioned oppositely (**L**). Additional statistical information was provided in Table S3.

Typically, the induction of EMT promotes the progression of CRC and correlates with the worse prognosis of CRC patients [17]. As a downstream effect of EMT, the cytoplasm-nucleus shuttling of TET2, which inhibits CRC progression, has been reported in our previous study [12]. To untwist this controversy, the growth and EMT of SW620 were monitored in depth during the LTC assay (Fig. 4A).

During the LTC assay, the percentage of SW620 cells outside colonies gradually increased from day 6 to day 15 (Fig. 4B). Interestingly, the proliferation rate of SW620 cells was faster from day 6 to 9 compared to day 12 to 15 overall (Fig. 4C). However, upon separating and analyzing the cells inside and outside colonies, we discovered that the cells inside colonies proliferated much more slowly than those outside from day 12 to 15 (Fig. 4C).

Next, we investigated how EMT regulates the proliferation and migration of SW620 cells during the LTC assay. EMT and its reversal process, mesenchymal-epithelial transition (MET), were induced by TGF-β and RepSox, respectively [18]. TGF-β suppressed cell growth, whereas RepSox facilitated the cell proliferation (Fig. 4C). Furthermore, TGF-β promoted the migration of cells out of colonies (Fig. 4D, E), and a higher percentage of cells outside colonies by day 15 was observed (Fig. 4F). Given that the cells outside colonies proliferated faster than those inside (Fig. 4C), the migration observed following TGF-β treatment was associated with an overall increase in cell numbers by day 15 (Fig. 4C–F). Conversely, RepSox inhibited migration but did not reduce the overall cell number significantly (Fig. 4C–F). The diminution in the overall cell population after RepSox treatment may not be evident until longer-term monitoring of cell proliferation is conducted.

The relationship between EMT and TET2 was assessed in this study. On the one hand, TGF-β-induced EMT increased the percentage of cells with nuclear TET2, whereas RepSox had the opposite effect (Figs. 4G and S4A), indicating that EMT correlates with the cytoplasm-nucleus shuttling of TET2. As SW620 cell colonies grow in a muti-layers pattern (Figs. S3A and S4B), it is difficult for the estimation of cell number via cell image, so cell colonies were disassociated into single cells and counted. Overexpression of TET2CD enhanced TGF-β‘s ability to suppress cell proliferation, while *TET2* knockout weakened this effect (Fig. 4H). This suggests that TGF-β-induced cytoplasm-nucleus shuttling of TET2 contributes to the inhibitory effects of TGF-β on cell proliferation, at least partially. However, overexpression of TET2CD and *TET2* knockout did not affect the actions of RepSox (Fig. 4H), possibly due to RepSox’s limited impact on the subcellular localization of TET2 in SW620 cells (Fig. 4G).

Given that TET-mediated DNA demethylation has been implicated in facilitating MET during somatic cell reprogramming [18], we further investigated how overexpression of TET2CD and *TET2* knockout influence cell migration. As shown in Fig. 4I–K, overexpression of TET2CD inhibited the cell migration out of colonies during days 12 to 15. Additionally, markers associated with EMT were suppressed by TET2CD on day 15 (Fig. 4L). Conversely, *TET2* knockout in SW620 cells was associated with the opposite effects (Fig. 4I–L), indicating an inhibitory role of TET2 in EMT. Collectively, a negative feedback loop exists between EMT and the cytoplasm-nucleus shuttling of TET2 (Fig. S4C).

Furthermore, we engineered TET2 constructs with artificial NES or NLS signals. When fused with an NES, TET2 remained cytoplasmic in SW480 cells, even under TGF-β. Conversely, when fused with an NLS, TET2 was constitutively localized in the nucleus in SW620 cells, regardless of EMT inhibition (Fig. S4D). Taken together, these findings demonstrate that the cytoplasmic-to-nuclear translocation of TET2 is independent of the classical NES/NLS system, and instead relies on its C-terminal domain [12], similar to the non-canonical mechanism of CTNNB1.

### Negative feedback between WNT activation and the nuclear shuttling of TET2

The interplay between EMT induction and the WNT activation has been extensively documented [9]. In our study, we verified this crosstalk in the LTC system. Treatment with RepSox and TGF-β, respectively was associated with decreased and elevated expression of the downstream target genes in WNT pathway (Fig. S5A). Similarly, modulation of the WNT pathway using its inhibitor, IWR1, and activator, IM12 [19], led to decreased and increased expression of EMT markers, respectively (Fig. S5B).

Moreover, IM12 increased the percentage of cells outside colonies on day 15 of LTC (Fig. S6A, B) and promoted the nuclear localization of TET2 (Fig. S6C). Conversely, IWR1 showed opposite effects (Fig. S6A–C), indicating that WNT activation is associated with the cytoplasm-nucleus shuttling of TET2 during CRC progression.

Furthermore, the downstream targets in the WNT pathway were suppressed by TET2CD on day 15 (Fig. 4L). Conversely, *TET2* knockout in SW620 cells enhanced the expression of these targets (Fig. 4L). Thus, a negative feedback loop also exists between WNT activation and the cytoplasm-nucleus shuttling of TET2. In addition, TET2 constructs with artificial NES or NLS signals failed to respond to WNT activation or WNT inhibition (Fig. S4D).

### ScRNA-seq analysis of SW620 cell colonies from different culturing time

**Fig. 5:**
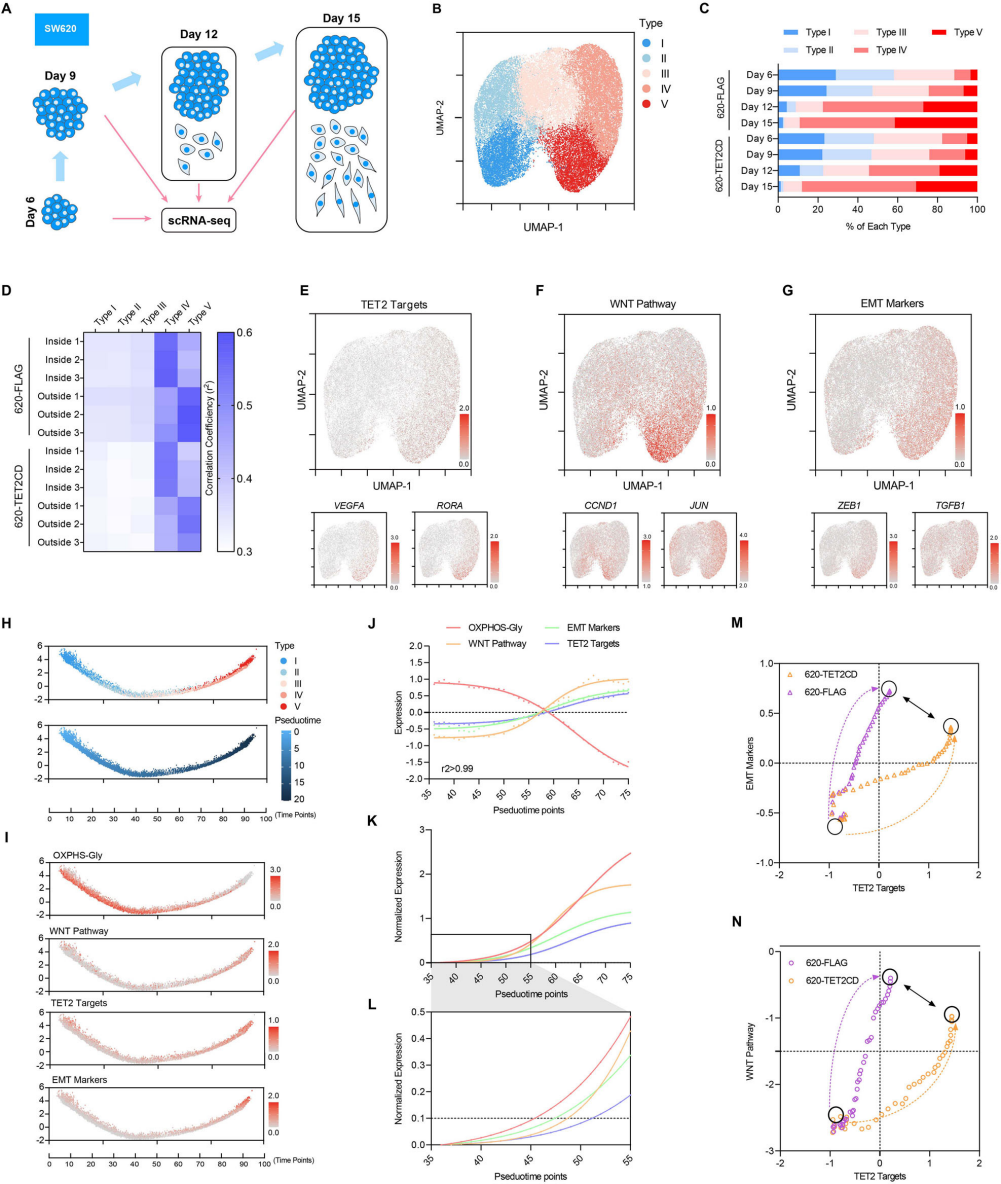
ScRNA-seq of SW620 LTC at different time points. **A** A schematic illustration of the scRNA-seq analysis of LTC. **B**, **C** UMAP for all 43,926 cells that passed the RNA quality control steps. The cells were clustered into five groups according to gene expression (**B**). The percentages of the five types of cells on days 6, 9, 12 and 15 were summarized (**C**). **D** The expression profiles of type IV and type V cells were close to those of cells inside colonies and outside the colonies, respectively. **E** UMAP plot of TET2 targets, which were identified in previous report [12]. **F** UMAP plot of WNT pathway targets, which were selected from KEGG 04310. **G** UMAP plot of EMT markers, which were reported previously [23]. **H**, **I** Pseudotime analysis of genes related to metabolism, the WNT pathway, the activation of TET2 targets and EMT. Genes related to OXPHOS and glycolysis were selected from KEGG 00010 & 00190. **J**–**L** Nonlinear regression was used to estimate the correlation between and the expression of indicated genes (**J**). The nonlinear regression results were normalized and compared to reveal the temporal sequence of expression changes (**K**, **L**). **M**, **N** The expression of genes related to the EMT/WNT pathways in type Ⅲ cells (pseudotime points 36–75) was plotted against those of TET2 targets. SW620-FLAG cells were plotted separately from those expressing TET2CD. Notably, TET2 and the EMT/WNT pathways formed negative feedback loops.

We also conducted scRNA-seq analysis on the cells harvested on day 6, 9, 12, and 15 (Fig. 5A). More than forty thousand SW620 cells expressing FLAG or TET2CD were sequenced and classified into 5 types (Types I, II, III, IV, and V) (Fig. 5B). The percentage of Type I cells gradually decreased during LTC, while the percentage of Type IV/V cells increased, indicating a progressive transition from Type I to Type V (Figs. 5C and S7A). The overexpression of TET2CD impaired this transition, as evidenced by a reduced percentage of Type V cells in SW620-TET2CD (Fig. 5C).

Transcriptome analysis suggested that Type IV and Type V cells corresponded to cells inside and outside the colonies, respectively (Figs. 5D and S7B). The ability of TET2CD to decrease the percentage of Type V cells further confirmed its role in suppressing EMT and the proliferation of cells outside the colonies.

Markers specific to these five cell types were identified and subjected to Gene Ontology (GO) analysis. These markers were associated with processes such as hypoxia, EMT, extracellular matrix dynamics, metabolism, cell cycle regulation, and WNT pathway signaling (Fig. S7C). Additionally, TET2 target genes such as *VEGFA* and *RORA* showed higher expression in Type V cells (Fig. 5E), correlating with the increased expression of WNT pathway targets *CCND1* and *JUN*, as well as EMT markers *ZEB1* and *TGFB1* (Fig. 5F, G).

Then, the cells from the LTC scRNA-seq were divided into 100 groups across a pseudotime course (Fig. 5H). The expression profiles of genes associated with metabolic switching (oxidative phosphorylation and glycolysis), WNT pathway, EMT, and TET2 were plotted along this pseudotime trajectory (Fig. 5I). Nonlinear regression analysis revealed that the expression of metabolic gene changed earliest, followed by alterations in the WNT pathway and EMT-related genes, with the TET2 target genes changed last (Fig. 5J–L). This temporal sequence outlined an event timeline starting from metabolic reprogramming through EMT induction/WNT pathway activation and concluding with TET2 translocation. However, the observed temporal sequence “metabolic changes-EMT/WNT activation-TET2-related events” reflects a “putative regulatory order” rather than a confirmed causal cascade. Further studies are still required to clarify the specific mechanisms underlying the crosstalk between these processes.

To validate the feedback loop between TET2 and EMT/WNT pathway described in Fig. 4, SW620-FLAG and SW620-TET2CD cells were analyzed separately along the pseudotime trajectory based on the expression of WNT pathway targets, EMT markers, and TET2 targets. Figure 5M demonstrates a positive correlation between the expression of TET2 targets and EMT markers in both SW620-FLAG and SW620-TET2CD cells, indicating the reciprocal relationship where EMT facilitates nuclear shuttling and activation of TET2. However, SW620-TET2CD cells exhibited greater upregulation of TET2 targets and less upregulation of EMT markers compared to SW620-FLAG (Fig. 5M), suggesting TET2’s inhibitory effects on EMT. Similar trends were observed when comparing the expression of WNT pathway targets and TET2 targets (Fig. 5N).

### In vivo validation of the negative feedback between EMT/WNT activation and the nuclear shuttling of TET2

ScRNA-seq was also performed on the SW620-transplanted nude mice models to validate the previous findings in vivo. Five distinct cell types (Fig. S8A) were identified, showing significant similarity to those observed in vitro (Fig. S8B). Based on the distribution of these cell types, the SW620 cells isolated from nude mice were suggested to resemble cells grown in dish during LTC assays (Fig. S8C).

Similar patterns of upregulated downstream targets in the WNT pathway, EMT markers, and TET2 targets were observed in type V cells and some type IV cells (Fig. S8D–F). Pseudotime analysis further confirmed a timeline starting from metabolic reprogramming to WNT activation and EMT induction and concluding with TET2 translocation (Fig. S8G–K).

Based on the expression of TET2, SW620 cells were classified into two groups: TET2-high and TET2-low. These groups were then independently analyzed along a pseudotime trajectory based on the expression of WNT pathway targets, EMT markers, and TET2 targets, akin to the analysis in Fig. 5M, N. This analysis revealed a similar feedback loop between WNT activation/EMT induction and TET2 activation (Fig. S8L, M). Furthermore, we did not observe any metastasis in this model. This model is focused on early stage of metastasis and invasion. However, future orthotopic transplantation models or genetically engineered mouse models (GEMMs) are still needed to illustrate the full process of tumor metastasis.

In addition, we expanded our experiments to additional cell lines (A549 and HepG2) for both in vivo and in vitro studies. For in vivo assays, A549 cells were used. Consistent with CRC cell lines, TET2 localized to the cytoplasm during early tumor growth (week 4) and translocated to the nucleus at later stages (week 6). As HepG2 cells fail to grow subcutaneously in nude mice in line with the description of HepG2 in ATCC. So we take SW480 cells (colorectal cancer) instead which served as negative control with most cells exhibiting nucleus TET2 at all time points (Fig. S9A). In vitro, SW620 cells naturally form compact colonies which mimic natural local tumor growth and onset a series of microenvironmental changes in the inside cells. But A549 and HepG2 cells grew as typical monolayers, which differed markedly from SW620 cells, they do not spontaneously form compact colonies (Fig. S9B). As the activation of WNT/EMT pathway is the most importent change of inside cells during colonies growth, so monolayer cells were treated with WNT/EMT pathway activators to mimic this microenvironment. Under these conditions, cells displayed nuclear TET2, consistent with in vivo observations (Fig. S9C).

### The clinical relevance of TET2 subcellular localization dynamics in CRC Progression

**Fig. 6:**
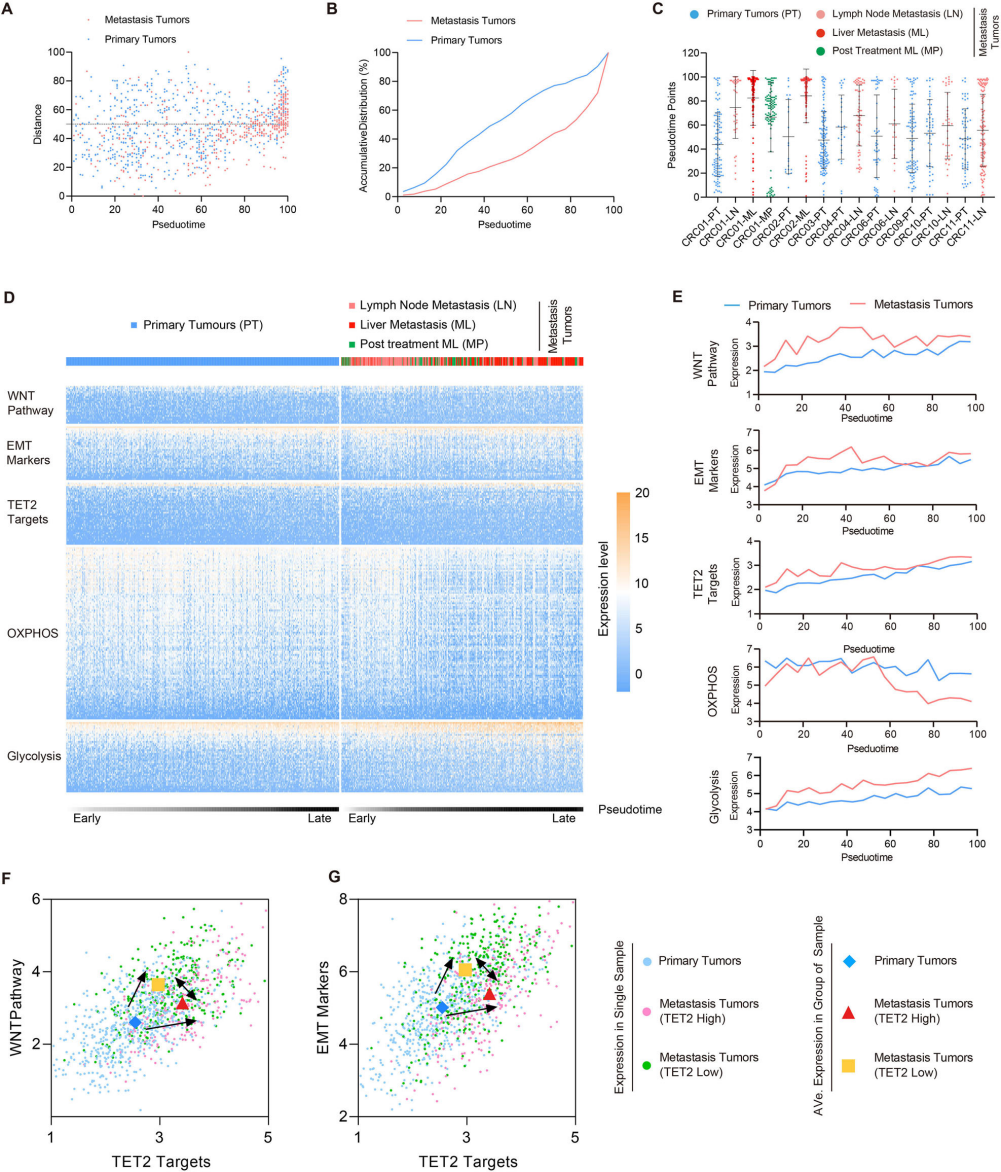
The LTC of SW620 cells resembles the progression in CRC patients. The scRNA-seq data (GSE97693) were plotted against the pseudotime course from the SW620 LTC (**A**) and the accumulative distribution was summarized in (**B**). **C** Cells from different patients were independently plotted against the pseudotime course. The expression of metabolic genes (OXPHOS to glycolysis), WNT pathway genes, TET2 targets and EMT markers were analyzed in heatmap (**D**) and curve graph (**E**). The expression of genes related to the WNT pathways (**F**) and EMT (**G**) was plotted against those of TET2 targets. Cells from MTs were separated into two groups based on the expression of TET2. Average expression in three groups of cells were plotted and connected with arrows. Additional statistical information was provided in Table S3.

To further validate the negative feedback loops between the EMT/WNT pathways and TET2 in clinical CRC, the scRNA-seq data of 1188 cells across 8 patients were analyzed alongside the pseudotime course derived from the SW620 LTC assays (Fig. 6A). Compared with cells from metastatic tumors (MTs), those from primary tumors (PTs) tended to cluster towards earlier pseudotime points (Fig. 6B, C). Key observations included the metabolic switching from oxidative phosphorylation (OXPHOS) to glycolysis, the activation of the WNT pathway, the upregulation of TET2 targets, and the induction of EMT in cells from later pseudotime points compared with the earlier ones (Fig. 6D). Notably, these changes were more pronounced in cells from MTs compared to those from PTs (Fig. 6E), underscoring their clinical relevance.

Based on the expression of TET2, MT cells were further classified into “TET2-high” and “TET2-low” types. Cells from PTs, MTs-TET2-high and MTs-TET2-low were plotted based on the expression of TET2 targets, WNT pathway genes and EMT markers. Moreover, cells in the “TET2-high” group exhibited lower expression levels of EMT markers and WNT targets, whereas cells in the “TET2-low” group showed higher expression levels, supporting the inhibitory role of TET2 in EMT induction and WNT activation (Fig. 6F, G).

To further confirm these findings clinically, 512 colon cancer samples from the TCGA dataset were analyzed similarly. Samples from stages I and II were predominantly plotted in early pseudotime, while samples from stages III and IV were more frequently in late pseudotime. Samples at late pseudotime exhibited metabolic switching, WNT activation, TET2 activation, and EMT induction, correlating with shorter overall survival (Fig. S10A–C). Similar relationships were observed among samples in the early pseudotime, late pseudotime with high TET2 expression, and late pseudotime with low TET2 expression, confirming the interplay between TET2, the EMT/WNT pathways, and clinical outcomes. Additionally, high TET2 expression in samples at late pseudotime was correlated with better survival of patient (Fig. S10D, E). Similar correlations were validated in 187 rectal cancer samples from the TCGA dataset (Fig. S11).

These analyses collectively support the role of TET2 in modulating the EMT/WNT pathway dynamics during CRC progression and highlight its clinical relevance in patient outcomes.

## Discussion

In this study, we found that the translocation of TET2 between the cytoplasm and nucleus is a gradual process rather than a binary event, potentially regulated by various factors and signaling pathways in the tumor microenvironment. In CRC samples, nuclear TET2 was predominantly found at the bottom of the mucosa, a crucial site for proximal metastasis initiation. The preferential localization of nuclear TET2 at the mucosa base that it may play a role in the early stages of metastasis.

In our previous study, we have demonstrated that EMT induction and WNT activation increased the nuclear localization of TET2, which is related to tumor suppression [12]. This is quite controversial as EMT induction and WNT activation are typically considered to promote tumor progression. EMT/WNT signaling is not uniformly pro-tumorigenic. Instead, its effects are highly context-dependent and, in certain settings, may even act in an opposing manner [20, 21]. To further clarify the controversial relationship between EMT/WNT signaling and TET2, as well as to address the inherent complexity of EMT/WNT pathway functions, we conducted both in vitro and in vivo studies. Due to the migration process of SW620 out of colonies resembles the CRC progression in both nude mice model and clinical patients; we designed the LTC assay to simulate tumor growth in vitro. Mouse tumor progression model of SW620 was also used in vivo. We found that the cytoplasm-nucleus shuttling of TET2 or the activation of TET2 is associated with EMT/WNT pathway activation and TET2 acts as a “side effect” or brake on EMT induction and WNT activation by forming a negative feedback loop with EMT/WNT pathway during tumor progression. The aforementioned conclusions are likewise evident in CRC tissue samples, and further investigations are imperative to elucidate the underlying mechanisms.

The CMS classification system is widely recognized as the most robust and biologically interpretable framework. Our analysis of multi-omics samples revealed that the observed phenomena are prevalent across CRC CMS subtypes, with CMS2, CMS4, and CMS3 (characterized by WNT/EMT dysregulation and metabolic alterations) accounting for the largest proportions. This highlights TET2’s critical role in coordinating epigenetic modulation with these oncogenic pathways, suggesting its important roles in tumor progression and metastasis across diverse molecular contexts and serves as a central node linking subtype-specific signaling, metabolic reprogramming, and CRC aggressiveness.

Extending beyond SW620 cells, we validated a conserved TET2-EMT/WNT feedback loop across multiple cell lines and cancer types. The localization and function of TET2 vary by cellular background and regulated by EMT/WNT pathway. Clinically, TET2 acts as an intrinsic tumor suppressor, holding therapeutic potential. Our prior in vivo/in vitro studies showed combining TET2 activator vitamin C (Vc) with EMT/WNT activators significantly reduced PDX tumor growth and lowered the effective Vc dose.

Since our SW620 LTC model only mimics local tumor growth. Future studies using orthotopic transplantation models or GEMMs are needed to clarify TET2’s roles throughout tumor initiation, progression, and distant metastasis. In summary, our findings revealed that the EMT/WNT pathway exerted certain tumor suppressive effects through inducing the cytoplasm-nucleus shuttling of TET2 and its subsequent activation. Collectively, these data imply that TET2 activation functions as an intrinsic brake on cancer progression, and represents a crucial therapeutic target for tumors.

## Methods

The methods used in this study are listed in the Supplementary Methods and Materials.

### Materials

The materials and reagents used in this study are listed in Supplementary Table S2.

### Statistical analysis

All experiments were replicated for five times with the exception of sequencing experiments. For animal studies, each group included at least 5 mice. Samples were randomly assigned to experiment groups. Experiments data were analyzed in GraphPad Prism 8.0. Error bars and “*n*” represent the standard deviation (standard error only if indicated) and the number of independent experiments, respectively. “*”, “**”, and “***” represent significant differences (*P* < 0.05, *P* < 0.01, and *P* < 0.001, respectively) from the indicated control groups. Additional statistic information was listed in Supplementary Table S3.

## Supplementary information

Supplementary Materials

Graphical Abstract legend

Table S1

Table S2

Table S3

Original Western Blots

## Data Availability

The RNA-seq and scRNA-seq results generated in this study are available at Gene Expression Ominibus (GEO) under accession number GSE269621 [Secure token: qpsdyesijtwzpul] & GSE188329 [Secure token: ivknawwarxahbct]. The other published dataset (GSE97693, TCGA cancer datasets in UCSC Xena datasets) [22] used in the current studies were listed in Supplementary Table S2. This study did not generate code. Data are available on reasonable request.
