# Supplementary material for: Cytoplasm-nucleus shuttling of TET2: an intrinsic brake in colorectal cancer progression
Source: Cell Death Dis. 2026 Jan 28;17(1):163. doi: 10.1038/s41419-026-08418-5 (PMC12877129; doi:10.1038/s41419-026-08418-5)
Supplement: Supplementary file 1 — Supplementary Materials [file 41419_2026_8418_MOESM1_ESM.docx]

**Supplementary Materials**

**Cytoplasm-Nucleus Shuttling of TET2: An Intrinsic Brake in Colorectal Cancer Progression**

Changpeng Li^1,*,#^, Fei Meng^1,2,*^, Jingcai He^1,3,*^, Linna Dong^1,4,*^, Yuexian He^1^, Qing Guo^1^, Kerou Zeng^1^, Yanhua Wu^1,4^, Haofei Ge^1,5^, Shiyu Chen^1,4^, Tingting Yang^1,2,4^, Yusheng Zhou^1,2,4^, Yulu Wang^1,4^, Lin Liu^1,4^, Qiwen Ren^1,4^, Meiai He^1,3,4^, Hao Sun^1^, Lining Liang^1^, Lin Guo^1^, Xiaolin Li^6^, Jiahong Hong^6^, Zhenhua Huang^6,#^, Hui Zheng^1,2,4,5,#^

^1^Guangdong Provincial Key Laboratory of Stem Cell and Regenerative Medicine, Guangdong-Hong Kong Joint Laboratory for Stem Cell and Regenerative Medicine, GIBH-CUHK Joint Research Laboratory on Stem Cell and Regenerative Medicine, Guangzhou Institutes of Biomedicine and Health, Chinese Academy of Sciences, Guangzhou, 510530, China.

^2^Centre for Regenerative Medicine and Health, Hong Kong Institute of Science & Innovation, Chinese Academy of Sciences, Hong Kong SAR, China.

^3^Key Laboratory of Biological Targeting Diagnosis, Therapy and Rehabilitation of Guangdong Higher Education Institutes, The Fifth Affiliated Hospital of Guangzhou Medical University, Guangzhou 510799, China.

^4^University of Chinese Academy of Sciences, Beijing, 100049, China.

^5^Joint School of Life Sciences, Guangzhou Medical University, 511436, China.

^6^Department of Oncology, Nanfang Hospital, Southern Medical University, Guangzhou, 510515, China.

#Correspondence to: Hui Zheng, Zhenhua Huang, and Changpeng Li; Email: zheng_hui@gibh.ac.cn, happygao@smu.edu.cn, and li_changpeng@gibh.ac.cn. #190 Kaiyuan Ave. Science City, Huangpu District, Guangzhou, 510530, China. Phone&Fax: 86-20-32015334.

^*^ These four co-authors contributed equally to this work

**METHODS**

**Immunohistochemistry (IHC) and patients’ classification**

In our previous study [1], tissue arrays and paraffin sections of CRC samples were stained using antibodies against TET2 and 5mC. In simple terms, the slices were first treated by deparaffinization. Then, they were subjected to antigen retrieval (citrate buffer, pH 6.0) and had their endogenous peroxidase activity blocked (3% H_2_O_2_). Next, the primary antibodies were incubated overnight at 4 ℃. After that, the slices were washed with PBS and incubated with HRP-Goat Anti-Rabbit/Mouse Universal Recombinant Secondary Antibody. Finally, a color reaction was carried out using the DAB reagent kit. In the current study, we analyzed the immunohistochemistry (IHC) staining results by dividing the samples based on additional criteria. And the IHC intensity was scored by three experts blinded with background information to avoid subjectivity. In the present study, the whole slide was scanned with TissueFaxs System as representative slides shown in Figure1 and Figure S1. Note that, in our previous studies [1], CRC tissue arrays and paraffin sections with high TET2 expression were categorized into two groups: "high TET2 in the cytoplasm" and "high TET2 in the nucleus" based on the subcellular localization of TET2. For samples exhibiting both forms of subcellular localization, the predominant form (>50%) determined the final subcellular localization classification for the entire sample. 55.5% of the samples in the “high TET2 expression in nucleus” group exhibited pure nucleus localization and were classified as the “nucleus only” group. The other 44.5% of the samples showed a mosaic pattern with both nuclear and cytoplasm TET2 were considered as the “nucleus major” group. Similarly, 27.0% of the samples in the “high TET2 expression in cytoplasm” group equipped with a mosaic localization pattern were considered as the “cytoplasm major” group, while 73.0% of the samples with pure cytoplasm localization were considered as the “cytoplasm only” group (Figure 1A-B and Table S1).

All studies were approved by the Institutional Review Board of Guangzhou Institutes of Biomedicine and Health (No. GIBH-IRB07-2017).

Samples previously categorized as "high TET2 in the nucleus" were subdivided into the “nucleus only” and “nucleus major” groups based on the presence or absence of the cytoplasmic localization of TET2. Similarly, samples previously categorized as "high TET2 in the cytoplasm" were classified into the "cytoplasm only" and “cytoplasm major” groups based on the presence or absence of the nuclear localization of TET2. Samples from the "TET2 low expression" group were not included in the analysis due to their low TET2 intensity.

The tumor tissue areas adjacent to the colon matrix were defined as the bottom of the mucosa, while areas opposite the colon matrix were defined as the top of the mucosa.

The clinical information of these samples and semi-quantified IHC results were listed in Supplementary Table S1.

**Animals**

BALB/c^nu/nu^ mice were obtained from Beijing Vital River Laboratory Animal Technology. Mice were normally housed in groups of four per cage with access to food and water *ad libitum*. All animal assays were conducted in line with the National Institutes of Health Guide for the Care and Use of Laboratory Animals (NIH Publication No. 80-23) and all procedures were approved by the Institutional Review Board in Guangzhou Institutes of Biomedicine and Health (No.2019061). Efforts were made to minimize animal suffering and the number of animals used.

**Cell Lines**

Cancer cell lines were purchased from Cell Bank of Chinese Academy of Sciences (http://www.cellbank.org.cn/) and cultured in DMEM supplemented with 10% Fetal Bovine Serum (FBS, Excell), 1% nonessential amino acids (NEAA, Thermo Fisher), and 1% GlutaMAX (Thermo Fisher). Cells were maintained at 37 °C under 5% CO_2_. Cells used in this study were subjected to a mycoplasma test (MycoAlertTM, Lonza) to ensure that they were free of mycoplasma.

**Long-term cell culture (LTC)**

After the cells were digested and counted, 1×10^4^ cells were seeded in each well of a six-well culture plate and cultured until day 15. To separate the cells inside and outside colonies, medium removal, DPBS, and 5-min accutase digestion at 37℃ were carried out. The digested cell suspension was collected and separated using a 70 µm cell strainer (Catalog No. 352350, Falcon). The cells that passed through the cell strainer were single cells and were considered as the cells outside the colonies. The cell strainer was inverted back onto a 50 mL centrifuge tube, and the colony spheres were obtained by rinsing the strainer with DPBS. After centrifugation of the colony spheres at 250×*g* for 5 min, the cells were digested using 0.25% trypsin at 37℃ for 5 min and the digestion was terminated using MEF medium (DMEM+10% FBS). The cells inside the colonies were obtained by centrifugation at 250×*g* for 5 min, removal of the medium, and resuspension using DPBS.

**Mouse Xenografts Model**

Xenografts assay was conducted as previously described [1]. Briefly, 4 to 6-week-old BALB/c^nu/nu^ mice were randomly grouped and injected subcutaneously with 5×10^6^ logarithmically growing cells. Tumor sizes were monitored every 2 or 3 days with caliper [2]. Mice were sacrificed via CO₂ asphyxiation at the indicated time points in according to the procedure of AVMA. Place the mouse in the cage, secure the lid, and connect the CO₂ tube to the water bottle inlet. Open the cylinder valve to infuse CO₂ at 10%-30% of the chamber volume per minute until the cage is fully filled. Once the mouse collapses and is immobile, increase the gas flow (not exceeding 0.5 kPa). When the mice show no movement, no breathing, and dilated pupils, turn off CO₂ and observe for 2 minutes to confirm death. Xenografts were then collected for immunohistochemistry staining. During the mouse feeding, AkaLumine-HCl (3.3 mM, 0.1 mL) was administered by intraperitoneal injection to monitor the tumor growing via Xenogen IVIS spectrum Imaging System (PerkinElmer, Waltham, MA). The image data were analyzed with Living Image software packages (Version 4.5).

**Immunofluorescence (IF)**

Cells were fixed with 4% paraformaldehyde and blocked with blocking buffer PBS containing 10% normal goat serum (Beyotime, C0265), 1% bovine serum albumin (Genview, FA016) and 0.3% Triton X-100. Antibodies were diluted with blocking buffer and incubated with the samples for 12 hours at 4℃ followed by secondary antibody staining. Immunofluorescence was detected with a Zeiss LSM800.

**Colony Formation Assay**

Cells were plated in 12-well plates at a concentration of 800 cells/well and incubated for 15 days. Then cells were washed with PBS, fixed with 70% ethyl alcohol and stained with Crystal Violet. Colony sizes were measured via Image J software.

**Dot-Blot**

0.8 μL of genomic DNA (2-fold dilution, first concentration 1.25 μg/μL, six spots) was pipetted onto the nitrocellulose membrane. After drying and ultraviolet crosslinking, the levels of 5mC and 5hmC were measured via enhanced chemiluminescence reagents (Millipore, WBKLS0500).

**RNA-seq**

RNA was extracted from the indicated samples and RNA-Seq libraries were prepared using the TruSeq RNA Sample Preparation Kit v2 (RS-122-2001, Illumina). The sequencing was done with NextSeq 500 High Output Kit v2 (75 cycles) (FC-404-1005, Illumina) in accordance with manufacturer’s instructions. Gene Oncology analysis was conducted using DAVID 2021 (https://david-d.ncifcrf.gov/) [3].

**Single-cell RNA sequencing (scRNA-seq)**

The Chromium Next GEM Single Cell 3’ Reagent Kits v3.1 and the protocol from 10X Genomics were followed as recommended by the manufacturer's instructions as described. Briefly, colonies or tissues were dissociated into single cells and diluted in 1×phosphate-buffered saline (PBS) to 700-1200 cells per μL. A total of 66462 cells for *in vitro* cell culture samples and 26608 cells for *in vivo* nude mice samples with cell viability >90% were incorporated for 10X Genomics sc-RNA-SEQ. The cell suspension was loaded into the Chromium Next GEM Chip G and sorted in the Chromium Controller from 10X Genomics. The Cell-Gel Beads in Emulsion (GEMs) were then incubated to generate the barcoded cDNA. The cDNA was cleaned using Dynabeads and washed, followed by cDNA amplification and SPRI selection. The retrieved cDNA was enzymatically fragmented, end-repaired, poly-A tailed, and ligated. Size selection, adaptor ligation, and amplification were done. Sequencing was conducted using the HiSeq X Ten System (Illumina) according to 10X Genomics specifications. All quality control steps were carried out using the Qsep100 System (BIOptic) with High Resolution Cartridge, whereas the concentration was calculated using the Qubit 4.0 Fluorometer with Kit High Sensitivity assays.

The raw sequencing data were processed using the 10X Genomics Cell Ranger pipeline (version 4.0). Firstly, Illumina BCL files were de-multiplexed and converted to FASTQs using mkfastq. Secondly, the FASTQs were then used to quantify gene expression using Cell Ranger count and the GRCh38 (version refdata-gex-GRCh38-2020-A, 10X Genomics) human genome reference. Lastly, cell ranger aggr took the outputs from multiple runs of cell ranger count, and normalized these runs to the same sequencing depth and recomputed the gene-barcode matrices and analysis on the combined data.

Then we selected cells that had a mitochondrial read rate and expressed genes to filter out low-quality cells from our data set (gene numbers <200 or >4,500; total UMI counts <1,000 or >20,000; and percentage of mitochondrial genes >10). The count data of each cell was first scaled and then log transformed using the R package Seurat (v4.0.4). And the average sequencing depth is 44,919 reads/cell (800 Gb total data, paired-end 150 bp) for in vitro cultures and 57425 reads/cell (400 Gb total data, paired-end 150 bp) for in vivo nude mouse models. The normalized and scaled data were used for all downstream analyses. The Monocle 2 Pseudotime analysis was performed with five types of cells markers for the ordering gene’s function.

**Supplementary Table Legends**

**Supplementary Table S1. Cancer sample information in this study.**

[Excel]

**Supplementary Table S2. Materials used in this study.**

[Excel]

**Supplementary Table S3. Additional Statistic information in this study.**

[Excel]

**Supplementary Figures and Captions**

**Supplementary Figure S1**

**
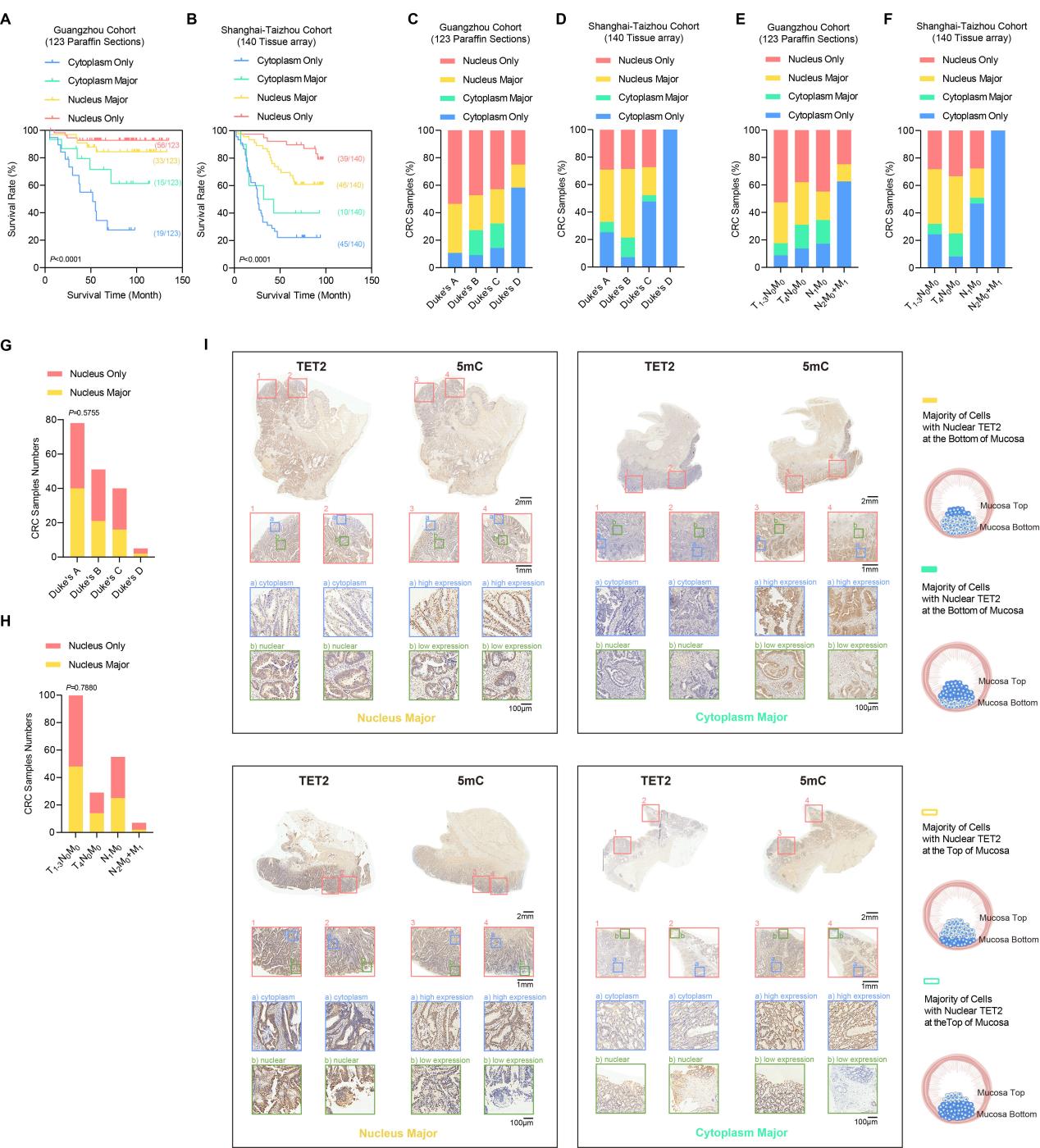
**

**Supplementary Figure S1. Nuclear TET2 is preferentially located at the bottom of the mucosa**

(A-B) The survival curves of patients in different groups. Guangzhou (A) and Shanghai-Taizhou cohort (B) were analyzed separately.

(C-F) Four groups of samples were analyzed with Duck’s (C-D) and TNM (E-F) staging system. Guangzhou (C&E) and Shanghai-Taizhou cohort (D&F) were analyzed separately.

(G-H) The percentages of “nucleus major” patients remained constantly across CRC stages.

(I) The representative IHC images for cells with nuclear TET2 at the bottom and at the top of the mucosa in the “cytoplasm major” and “nuclear major” groups.

Additional statistical information was provided in Table S3.

**Supplementary Figure S2**


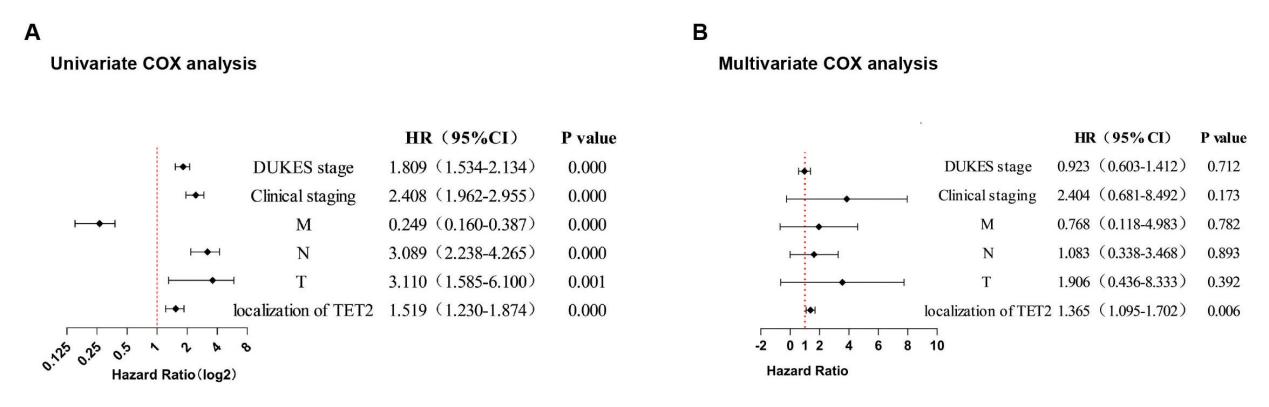


**Supplementary Figure S2. Univariate COX analysis and Multivariate COX analysis.**

1. Univariate COX analysis of variables included TET2 subcellular localization (nuclear vs. cytoplasmic) and key clinical factors. Significant variables were listed.
2. Multivariate COX analysis of confirmed that only TET2 subcellular localization remained an independent prognostic factor for OS (HR = 1.365, 95% CI: 1.095-1.702, p=0.006).

Additional statistical information was provided in Table S3.

**Supplementary Figure S3**


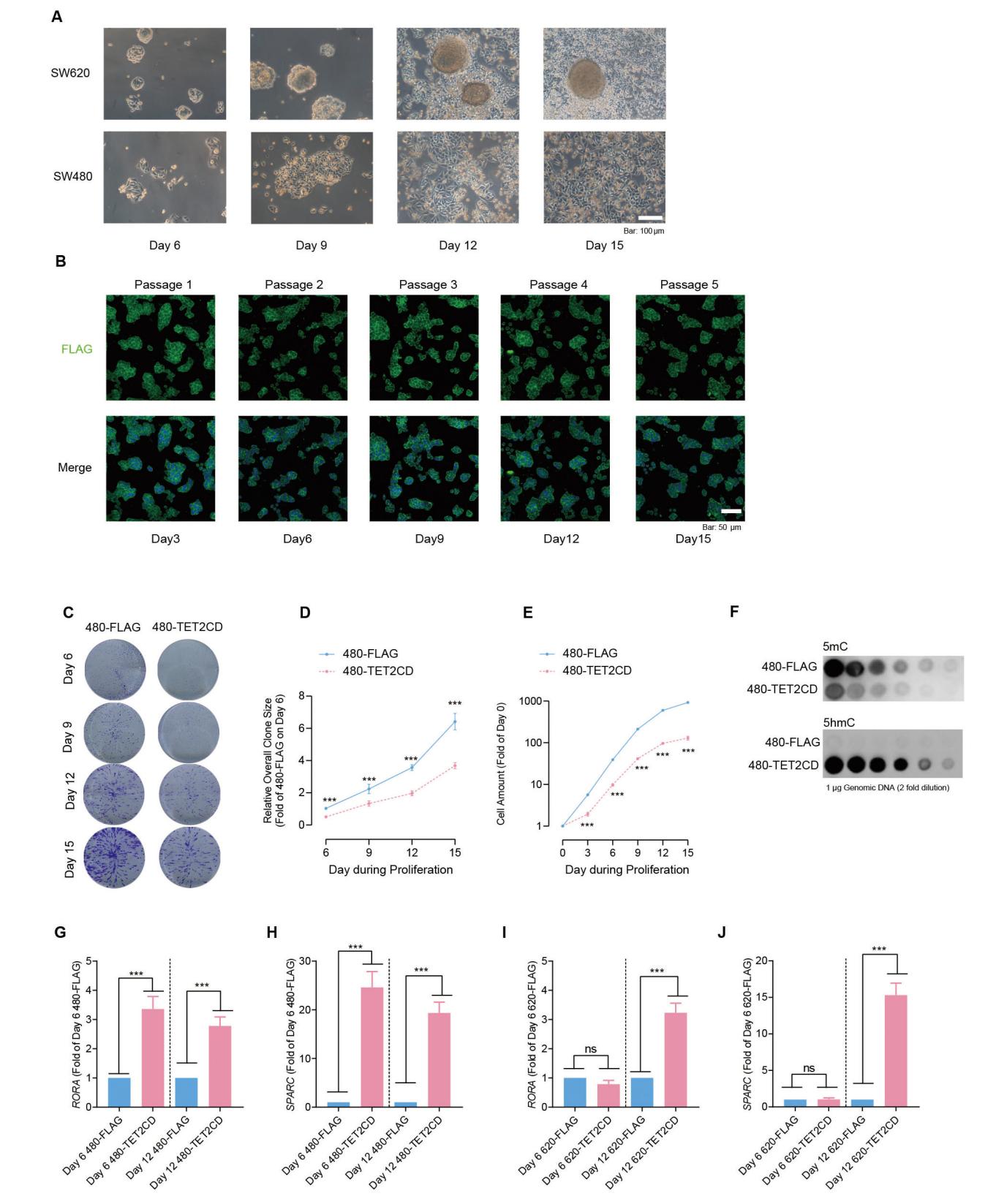


**Supplementary** **Figure S3. TET2 suppressed the growth of SW620 cells at the late stage of colony growth**

1. Phase contrast images of SW480 and SW620 cells during LTC
2. IF of SW620-TET2CD-Flag with regular passages.

(C-F) The colonies of SW480 were stained with crystal violet (C). The overall colonies size (D), and the cell number were summarized (E). The activity of TET2 in SW480 was measured with 5mC/5hmC antibodies on day 15 (F).

(G-J) The expression of *RORA* (G&I) and *SPARC* (H&J) were measured on Day 6 and Day 12 during the LTC of SW480 (G-H) and SW620 (I-J) cells.

Additional statistical information was provided in Table S3.

**Supplementary Figure S4**


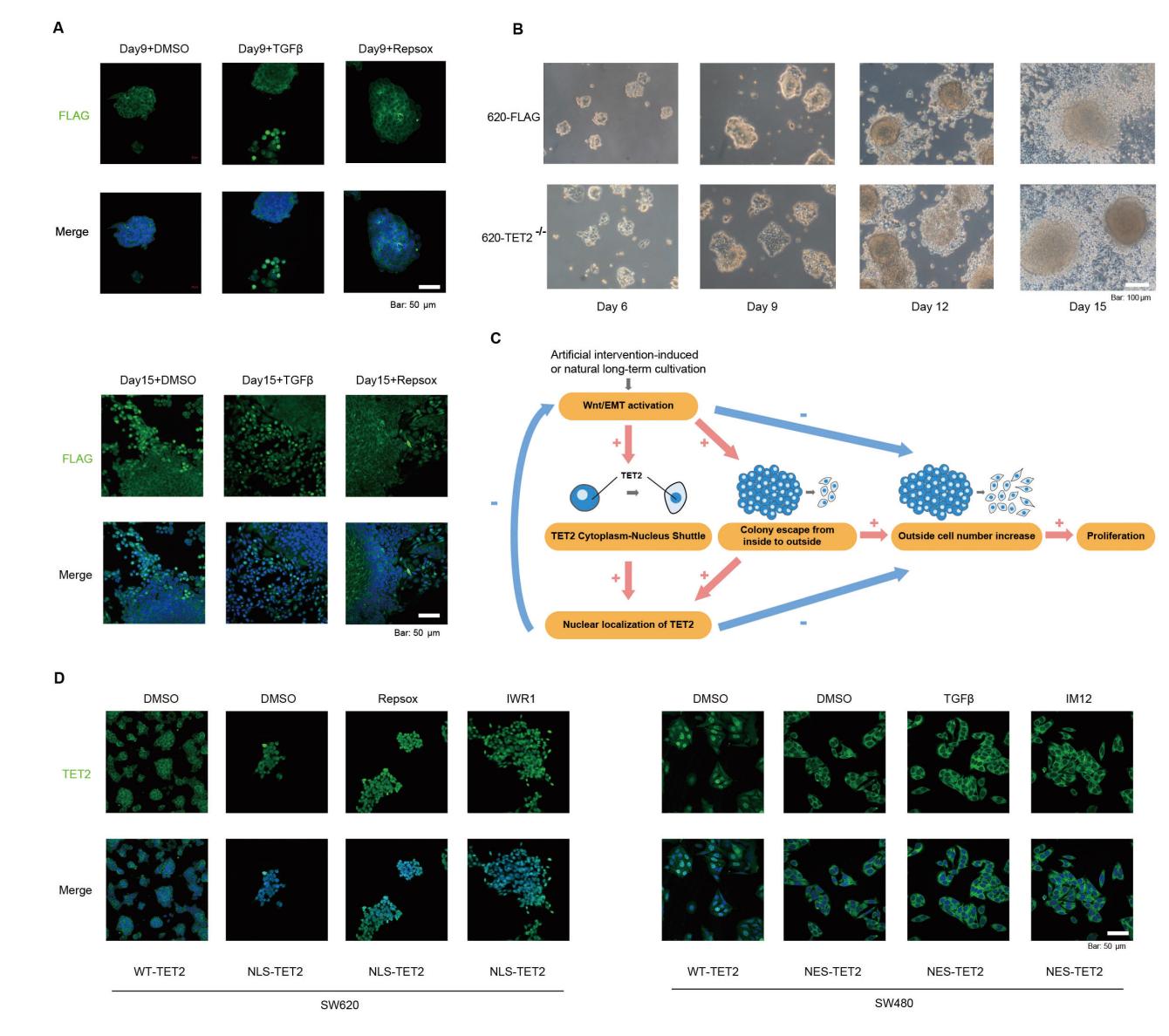


**Supplementary Figure S4. The impact of EMT and WNT pathway on the migration and TET2 localization of cells indicated.**

1. IF analysis of Day9 and Day15 colonies shows distinct TET2 localization with different treatments.
2. Long term culturing of TET2 knocked out SW620.
3. Schematic diagram shows the feedback regulation of EMT/WNT pathway and TET2 localization.
4. IF analysis of TET2CD localization in SW480 with WT-TET2/NES-TET2 and SW620 with WT-TET2/NLS-TET2 overexpression.

Additional statistical information was provided in Table S3

**Supplementary Figure S5**


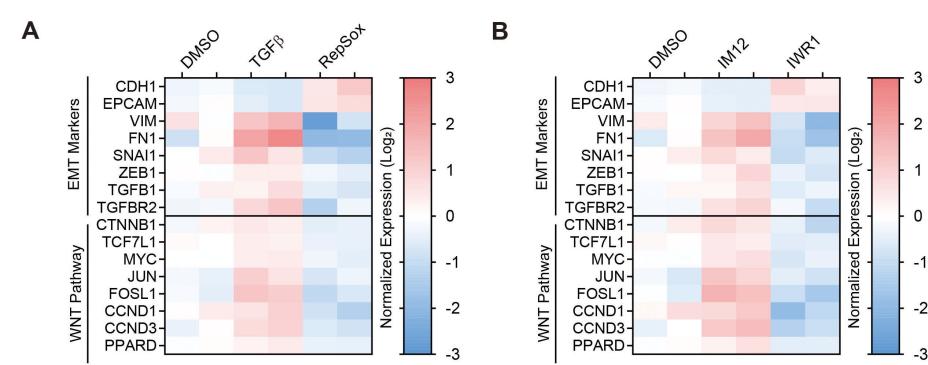


**Supplementary Figure S5. The interaction of the EMT and WNT pathway measured by RNA-SEQ**

(A) The expression of EMT markers and targets in the WNT pathway were summarized in SW620 cells treated with 1ng/mL TGF-β or 1 μM RepSox.

(B) The expression of EMT markers and targets in the WNT pathway was summarized in SW620 cells treated with 3.8 μM IM12 or 25 μM IWR1.

Additional statistical information was provided in Table S3

**Supplementary Figure S6**


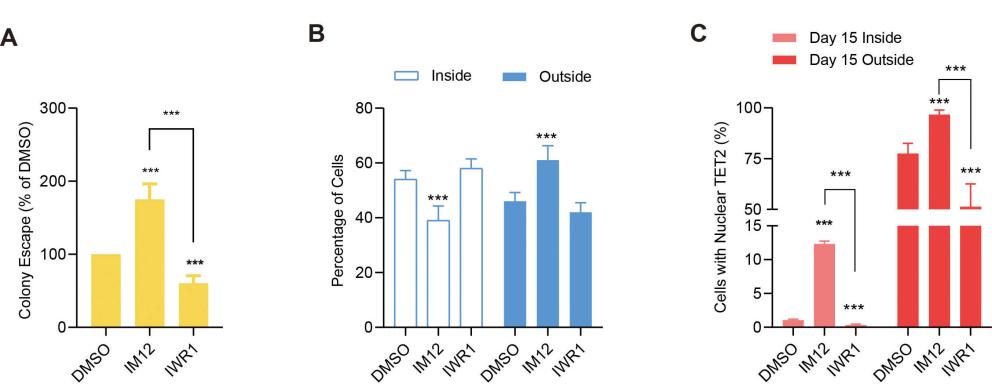


**Supplementary Figure S6. Modulating the WNT pathway affected the subcellular localization of TET2**

SW620 cells were treated with DMSO, 3.8 μM IM12 and 25 μM IWR1. The migration of cells out of colonies was calculated based on the proliferation rates and presented in (A). The percentages of cells inside and outsides the colonies on day 15 were summarized in (B). The percentages of cells with nuclear TET2 were listed in (C).

Additional statistical information was provided in Table S3.

**Supplementary Figure S7**


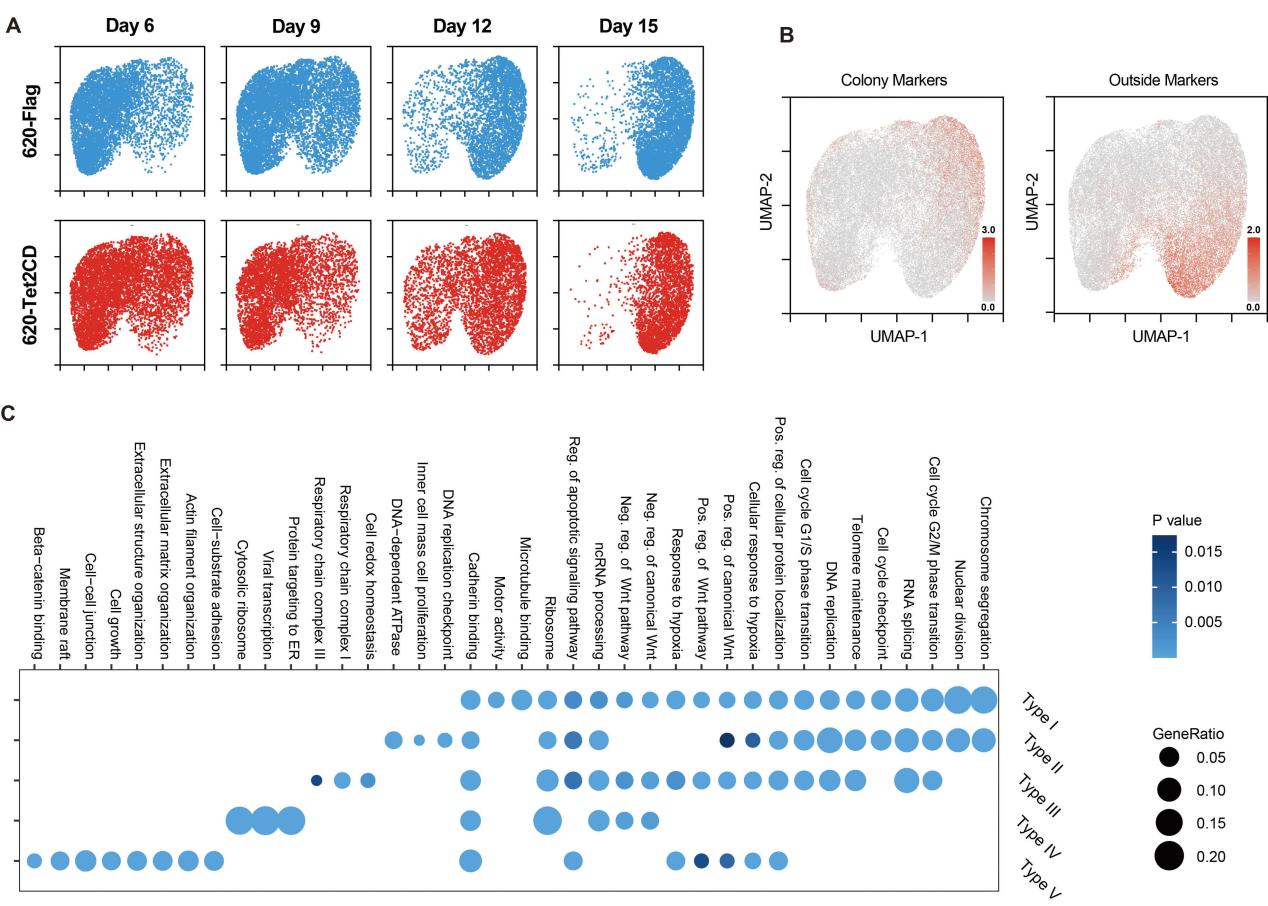


**Supplementary** **Figure S7. Cytoplasm-nucleus shuttling of TET2 during CRC progression**

(A) Cells on days 6, 9, 12 and 15 were summarized in UMAP plots.

(B) UMAP plot of the markers of cells inside and outside the colonies.

(C) GO analysis of the marker genes for the five types of cells.

Additional statistical information was provided in Table S3

**Supplementary Figure S8**


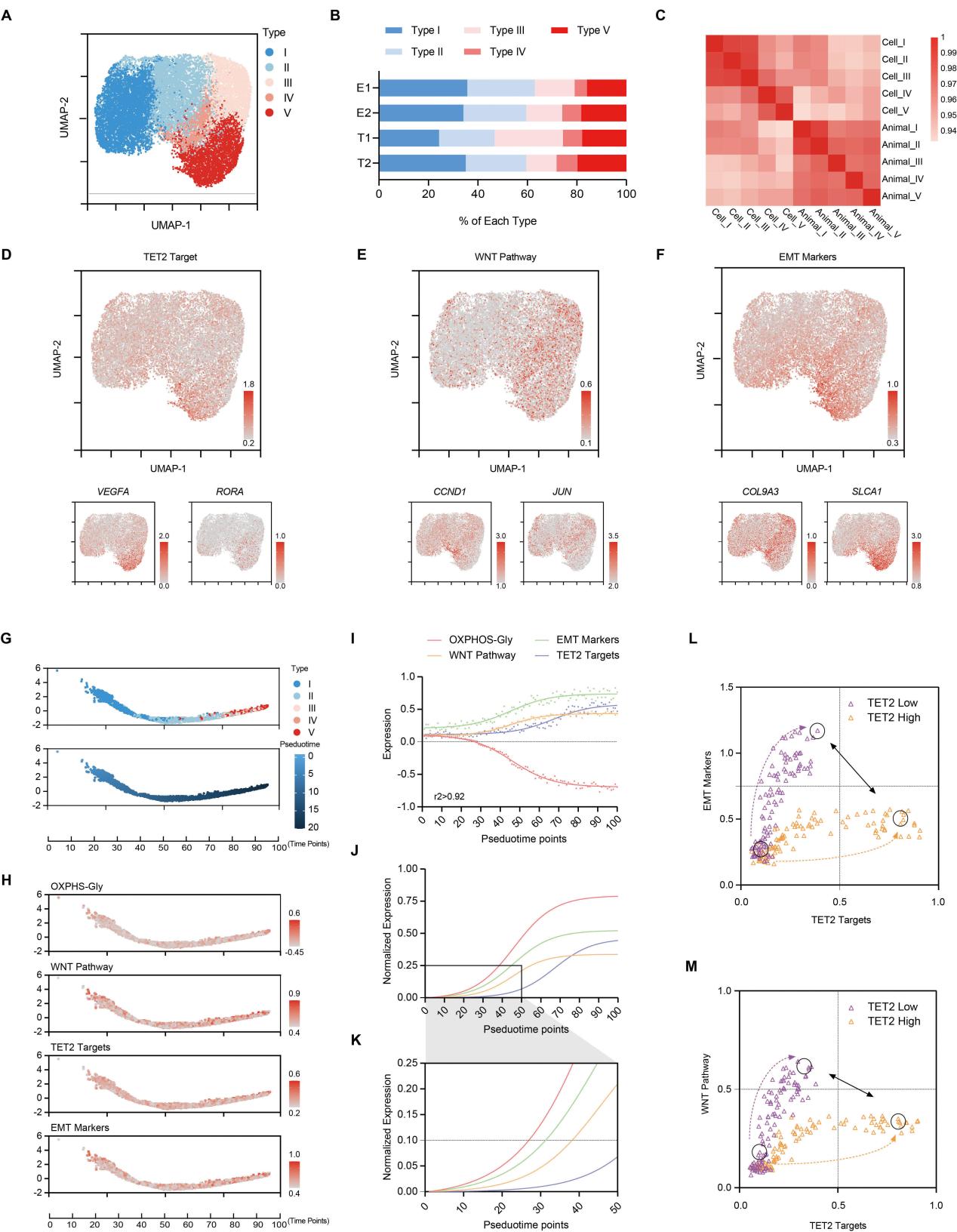


**Supplementary Figure S8. ScRNA-seq of the SW620-transplantaed nude mice model.**

(A-C) UMAP for all tumor cells that passed the RNA quality control steps. The cells were clustered into five groups according to gene expression (A). The percentages of the five types of cells were summarized in (B). E1, E2, T1, and T2 represent different mice tested. The expression profiles of the five types of cells were similar to those in Figure 5B (C).

(D-F) UMAP plot of TET2 targets (D), WNT targets (E), and EMT markers (F).

(G-H) Pseudotime analysis of genes related to metabolism, the WNT pathway, the activation of TET2 targets and EMT.

(I-K) Nonlinear regression was used to estimate the correlation between pseudotime and the expression of indicated genes (I). The nonlinear regression results were normalized and compared to reveal the temporal sequence of expression changes (J-K).

(L-M) The expression of genes related to the EMT/WNT pathways was plotted against those of TET2 targets. Cells were classified into two groups based on the expression of TET2.

Additional statistical information was provided in Table S3

**Supplementary Figure S9**


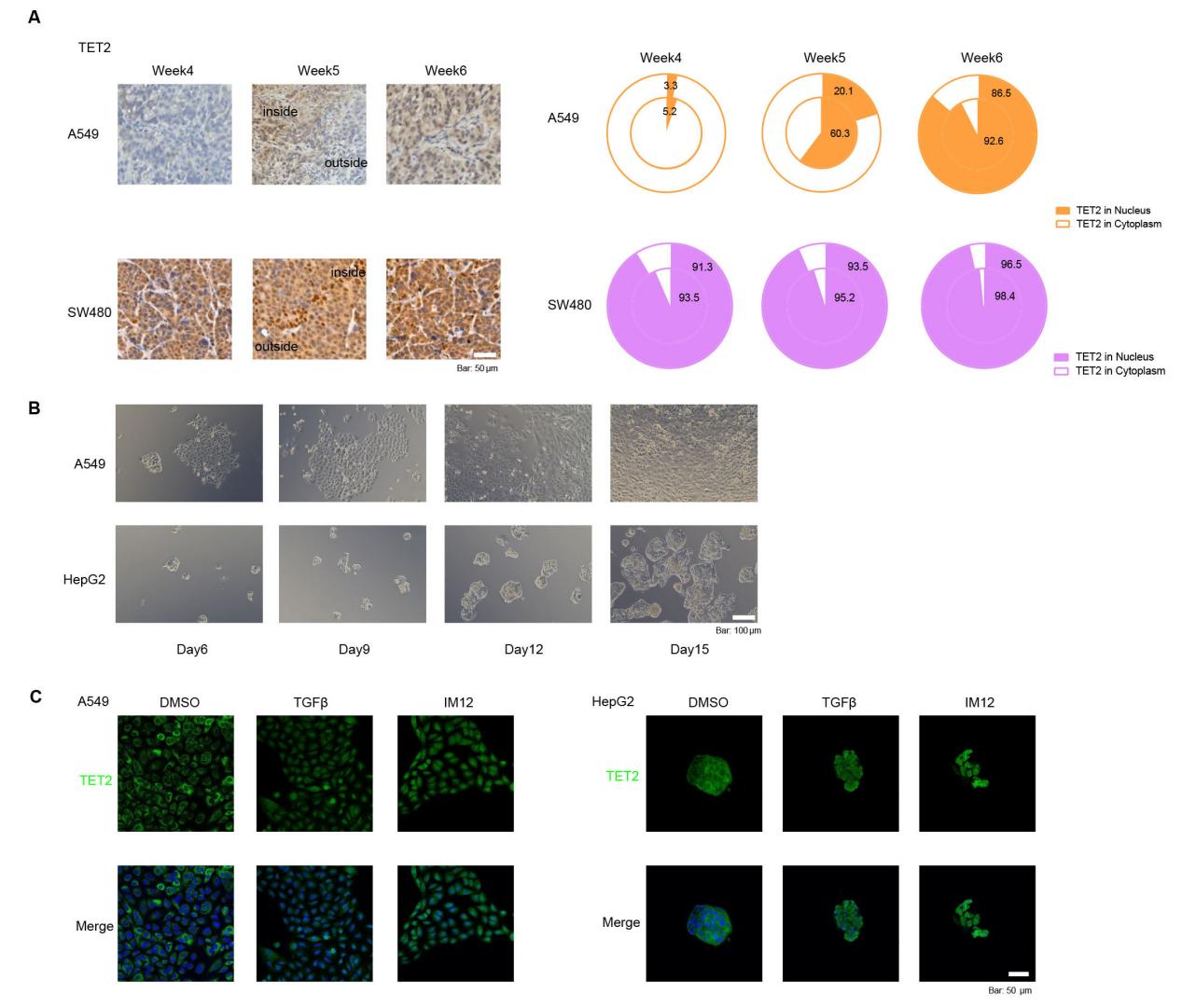


**Supplementary Figure S9. *In vivo* and *in vitro* analysis of TET2 localization changes in different tissue areas and different compounds treatments.**

1. *In vivo* nude mice subcutaneous tumor formation of A549 and SW480 at week4-6. The localization of TET2 was analyzed with IHC and related statistic summary was listed.
2. Phase contrast images of A549 and HepG2 cells during LTC.
3. IF analysis of TET2 localization changes with different compounds treatments in A549 and HepG2 cells.

Additional statistical information was provided in Table S3

**Supplementary Figure S10**


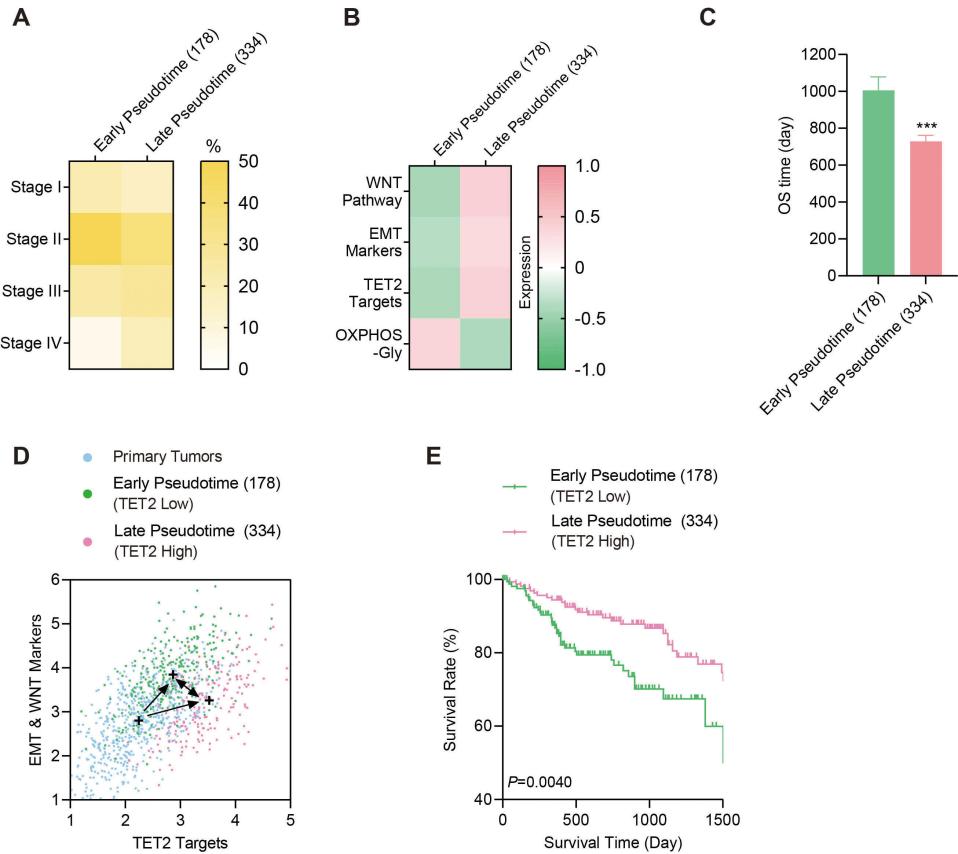


**Supplementary** **Figure S10. Pseudotime analysis of colon cancer TCGA samples**

(A) Colon cancer samples at different stages were classified into early and late pseudotime.

(B) The expression of EMT markers, WNT pathway targets, TET2 targets, and OXPHOS-glycolysis genes were summarized and compared between early and late pseudotime.

(C) The OS time of patients with colon cancer was compared between early and late pseudotime.

(D-E) The expression of EMT markers, WNT pathway targets, and TET2 targets were plotted. Samples were further divided based on the expression of TET2. The average expression in the three categories of cells was plotted and connected with arrows (D). The corresponding survival was analyzed with the Kaplan–Meier method (E).

Additional statistical information was provided in Table S3.

**Supplementary Figure S11**


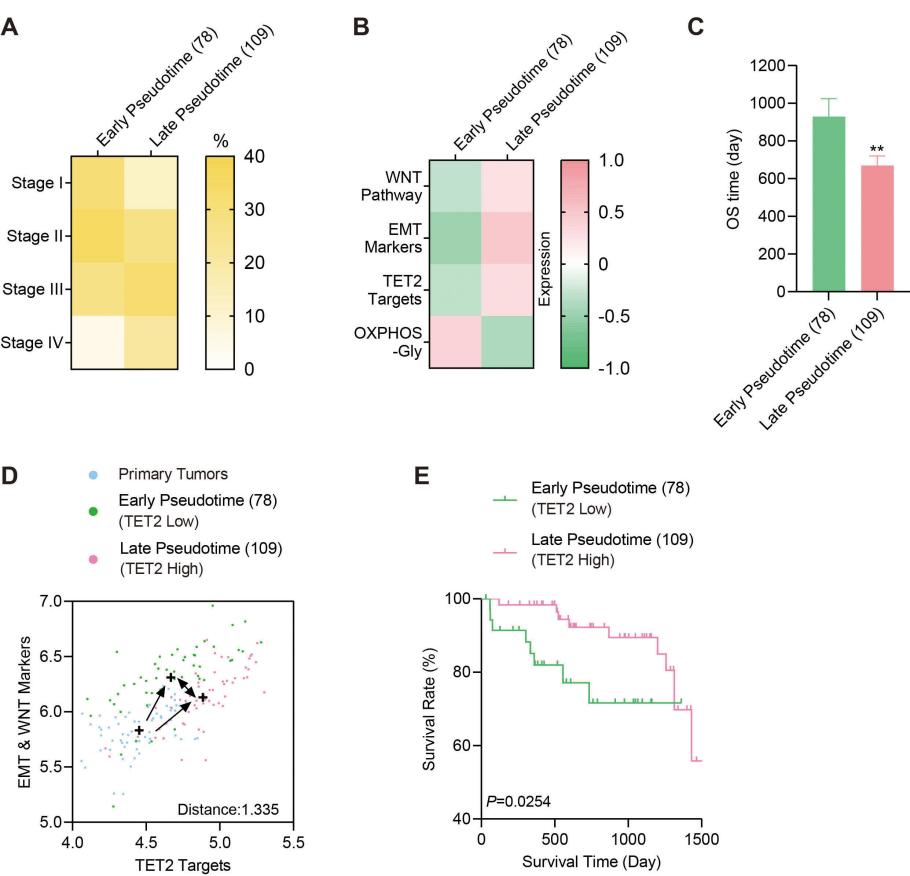


**Supplementary** **Figure S11. Pseudotime analysis of TCGA samples of rectal cancer**

(A) The rectal cancer samples at different stages were classified into early and late pseudotime.

(B) The expression of EMT markers, WNT pathway targets, TET2 targets and OXPHOS-glycolysis genes were summarized and compared between early and late pseudotime.

(C) The OS time of patients with rectal cancer was compared between early and late pseudotime.

(D-E) The expression of EMT markers, WNT pathway targets and TET2 targets was plotted. Samples were further divided based on the expression of TET2. The average expression in three categories of cells was plotted and connected with arrows (D). The corresponding survival was analyzed with the Kaplan–Meier method (E).

Additional statistical information was provided in Table S3.

**REFERENCES**

1 Li C, He J, Meng F, Wang F, Sun H, Zhang H, et al. Nuclear localization of TET2 requires beta-catenin activation and correlates with favourable prognosis in colorectal cancer. Cell Death Dis. 2023;14(8):552.

2 Iwano S, Sugiyama M, Hama H, Watakabe A, Hasegawa N, Kuchimaru T, et al. Single-cell bioluminescence imaging of deep tissue in freely moving animals. Science (New York, NY). 2018;359(6378):935-9.

3 Sherman BT, Hao M, Qiu J, Jiao X, Baseler MW, Lane HC, et al. DAVID: a web server for functional enrichment analysis and functional annotation of gene lists (2021 update). Nucleic Acids Res. 2022;50(W1):W216-W21.
