# Supplementary material for: Cytoplasm-nucleus shuttling of TET2: an intrinsic brake in colorectal cancer progression
Source: Cell Death Dis. 2026 Jan 28;17(1):163. doi: 10.1038/s41419-026-08418-5 (PMC12877129; doi:10.1038/s41419-026-08418-5)
Supplement: Supplementary file 2 — Graphical Abstract legend [file 41419_2026_8418_MOESM2_ESM.docx]

**Graphical Abstract TEXT**

Colorectal Cancer (CRC) progression is a complex and dynamic process closely linked to TET2-mediated mediated DNA demethylation. Nuclear increase of TET2 was observed at the invasion/migration front of CRC in clinical samples, CRC animal model and SW620 long term colony culture. Epithelial-mesenchymal transition (EMT) and the activation of the WNT pathway formed a negative feedback loop with TET2 which promote the nucleus localization of TET2 and tumor remission at the invasion front of CRC.
