# Supplementary figures and images for: Cytoplasm-nucleus shuttling of TET2: an intrinsic brake in colorectal cancer progression
Source: Cell Death Dis. 2026 Jan 28;17(1):163. doi: 10.1038/s41419-026-08418-5 (PMC12877129; doi:10.1038/s41419-026-08418-5)

Figure3 J

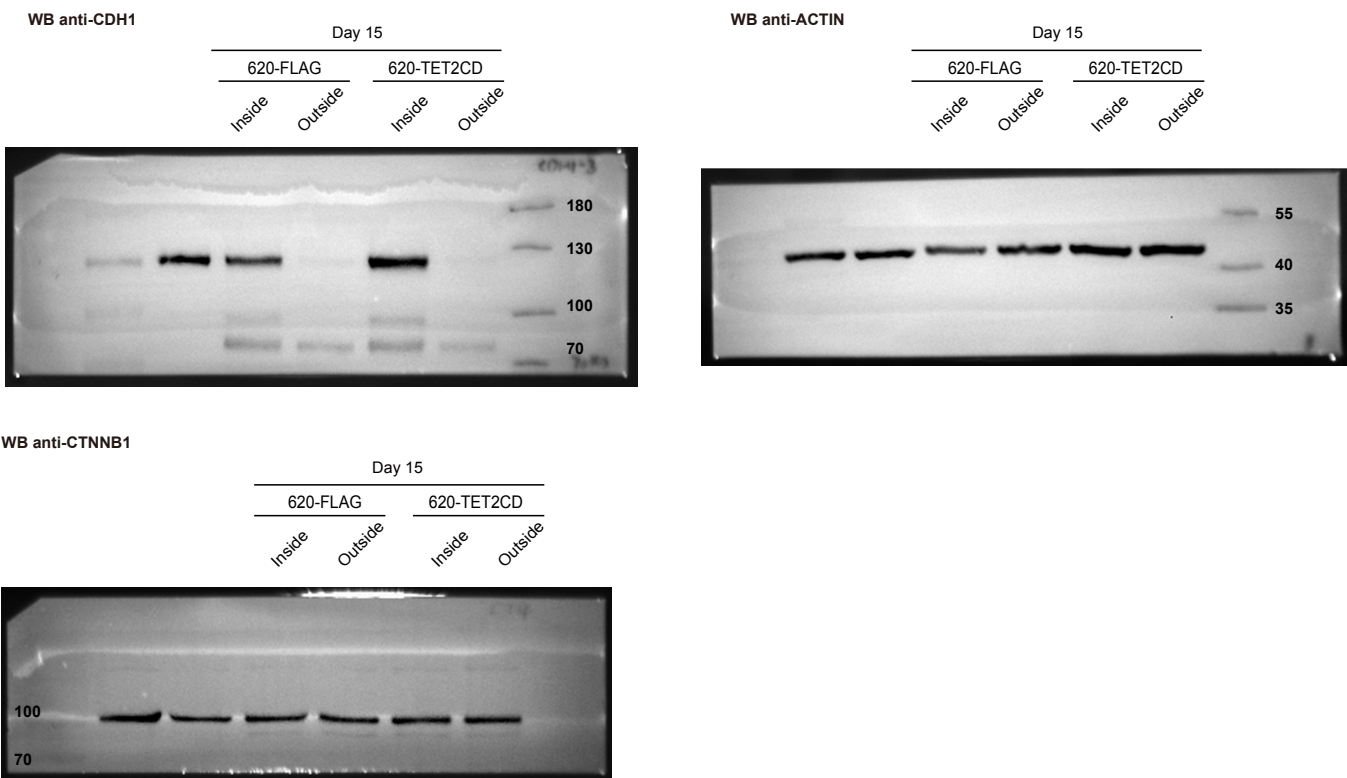

Supplement: Supplementary file 6 — Original Western Blots [file 41419_2026_8418_MOESM6_ESM.pdf]
